# Single‐Cell RNA‐Sequencing Reveals Cachectic Satellite Cell Population in Muscle of Male Mice With Cancer Cachexia

**DOI:** 10.1002/jcsm.70260

**Published:** 2026-03-27

**Authors:** Alex Brown, Nicolás Collao, Aisha Saleh, Natasha Strong, Michael De Lisio, Nadine Wiper‐Bergeron

**Affiliations:** ^1^ Graduate Program in Cellular and Molecular Medicine, Faculty of Medicine University of Ottawa Ottawa Ontario Canada; ^2^ School of Human Kinetics, Faculty of Health Science University of Ottawa Ottawa Ontario Canada; ^3^ Éric Poulin Centre for Neuromuscular Disease University of Ottawa Ottawa Ontario Canada; ^4^ Department of Cellular and Molecular Medicine, Faculty of Medicine University of Ottawa Ottawa Ontario Canada

**Keywords:** cancer cachexia, muscle atrophy, muscle satellite cells, single‐cell RNA sequencing, skeletal muscle

## Abstract

**Background:**

Cancer cachexia leads to decreases in body mass, lean mass and fat mass, decreased therapeutic potential and ~20% of cancer‐related deaths. While several studies have demonstrated changes to components of the muscle microenvironment with cancer cachexia, none have comprehensively assessed changes to cellular dynamics across the duration of cachexia development.

**Methods:**

Single‐cell RNA‐sequencing was performed on hindlimb muscles of male mice with 2‐, 2.5‐ and 3.5‐week subcutaneous Lewis‐lung carcinoma tumours. Cell population changes were confirmed with flow cytometry.

**Results:**

Body mass (−0.51 g; *p* = 0.0014) and lean mass (−0.85 g; *p* = 0.0134) were decreased at 2.5 weeks and were significantly lower than sham. Increases in fat mass were attenuated starting at 2 weeks (0.70 g; *p* = 0.0408) compared to sham (1.55 g), and muscle cross‐sectional area decreased at 3.5 weeks (−14.81%; *p* = 0.0022) compared to sham. We report a novel cachexia‐associated satellite cell subcluster, comprising 71.1% of the population at 3.5 weeks, corresponding with a +20.33% increase in cell size (*p* = 0.0266) and +19.73% increase in the proportion of activated PAX7^+^MYOD^+^ cells after 24 h cultured on individual myofibres (*p* = 0.0226). This cachexia‐associated subcluster was also present in C26 tumour‐bearing mice and had a unique gene expression signature compared to other muscle wasting disorders. The cachexia‐associated subcluster was enriched for signalling pathways (IL‐17, TNF, p53, NF‐κB, FoxO, adipocytokines, NOD‐like receptor, MAPK and JAK–STAT) implicated in satellite cell dysfunction in cancer cachexia. Prior to the emergence of cachexia‐associated satellite cells, increases in CD11b^+^ (+928.01%; *p* < 0.0001), Ly6C^low^ (+1080.85%; *p* < 0.0001), Ly6C^high^ (+920.33%; *p* = 0.0002), F4/80^+^CD206^−^ (+299.22%; *p* = 0.0039), F4/80^+^CD206^+^ (+1466.40%; *p* < 0.0001) immune cell populations were observed at 2 weeks compared to sham and returned to baseline by 2.5 weeks. There was also an increase in PDGFRα^+^ fibro‐adipogenic progenitors at 2 weeks (+53.44%; *p* = 0.0398) and decreased CD31^+^ endothelial cells at 2 weeks (−57.37%; *p* = 0.0014) and 3.5 weeks (−39.78%; *p* = 0.0213) compared to sham, with no change in ITGA7^+^ satellite cells (*p* = 0.4271). Cell communication analyses revealed a decline in cell communication with cancer cachexia in all cells except for monocytes/macrophages, and a decrease in cell adhesion‐related signalling in cachexia‐associated satellite cells, which is important for satellite cell differentiation, and may help to explain differentiation defects with cachexia.

**Conclusions:**

We describe a novel satellite cell subcluster unique to cachexia. We also identified increased immune cell and fibroadipogenic progenitor content and decreased endothelial cell content that precede muscle wasting with cancer, suggesting a role for these cell populations in satellite cell dysfunction and muscle atrophy in this condition.

**Abbreviations**
ANOVA: analysis of variance
bFGF: basic fibroblast growth factor
BSA: bovine serum albumin
CaSC: cachexia‐associated satellite cell
CSA: cross‐sectional area
DAPI: 4′6‐diamidino‐2‐phenylindole
DM: differentiation media
DMEM: Dulbecco's modified eagle medium
DS: Donkey serum
EDL: extensor digitorium longus
EdU: 5‐ethynyl‐2′‐deoxyuridine
ECM: extracellular matrix
FACS: fluorescence‐activated cell sorting
FAPs: fibro‐adipogenic progenitors
FBS: fetal bovine serum
GS: goat serum
GM: growth media
HGF: hepatocyte growth factor
HS: horse serum
ITGA7: integrin α7
LLC: Lewis‐lung carcinoma
MACS: magnetic‐activated cell sorting
MHC: myosin heavy chain
MuSCs: muscle satellite cells
MYOD: myogenic determination factor
PAX7: paired‐box transcription factor 7
PBS: phosphate‐buffered saline
PDAC: pancreatic ductal adenocarcinoma
PFA: paraformaldehyde
PDGFRα: platelet‐derived growth factor receptor α
scRNAseq: single‐cell RNA sequencing
vSMCs: vascular smooth muscle cells
WGA: wheat germ agglutinin

## 1 Introduction

Cancer cachexia is a complex disorder that affects 50%–80% of cancer patients, resulting in > 10% loss of body weight, decreased skeletal muscle and fat mass and is the cause of ~20% of cancer‐related deaths [1]. Muscle mass loss during cancer treatments decreases therapeutic effectiveness and patient survival [2]; therefore, maintaining muscle mass is essential for overall health, quality of life and is a key component in cancer survivorship. Because there are no currently approved therapeutic strategies that can stop or reverse cancer cachexia, it is necessary to identify early changes in skeletal muscle prior to cachexia induction to identify novel preventative approaches.

Skeletal muscle is comprised of several cell types that make up the microenvironment, including muscle fibres, resident stem cells (satellite cells; ‘MuSCs’), endothelial cells (capillaries and other blood vessels), fibroadipogenic progenitors (FAPs) for connective tissue and fat and immune cells, which respond to muscle damage to regulate repair [3]. Properly timed and coordinated communication between cells in the muscle microenvironment is essential for muscle maintenance and repair [3]. Several studies have described cancer cachexia‐induced changes to the muscle microenvironment [4, 5, 6, 7]; however, no study has comprehensively assessed cellular changes to the microenvironment over the course of cancer cachexia.

Single‐cell RNA sequencing (scRNAseq) technology has deepened our understanding of cell types and communication within the muscle [6, 8, 9, 10]; however, skeletal muscle cellular dynamics in several pathological conditions, such as cancer cachexia, have not been fully characterized. We combined scRNAseq and flow cytometry to identify early changes to the muscle microenvironment that precede muscle atrophy to identify cell types and pathways that may contribute to this pathology. Our findings revealed early alterations to cellular dynamics in skeletal muscle that precede cachexia development, resulting in a distinct cell fate of MuSCs unique to cancer cachexia.

## 2 Methods

Additional methods are in Supplementary Methods and Supplementary Tables for key resources (S1), immunofluorescence antibodies (S2), flow cytometry reagents (S3), magnetic‐activated cell‐sorting (MACS) antibodies (S4), software and algorithms (S5), R packages (S6) and complete blood cell counts (S7).

### 2.1 Animal Models

Animal procedures were performed according to the Canadian Council on Animal Care guidelines and approved by the Animal Care and Veterinary Services Committee at the University of Ottawa. Eleven‐week‐old C57BL/6 mice were purchased from Charles River Laboratories (Québec, Canada) and acclimatized for 1 week prior to experimentation. Male mice were used due to having more severe cachexia than females (Figure S1) [11]. Mice were housed individually with a 12‐h light/dark cycle and allowed food and water ad libitum (food intake in Figure S2). Mice were euthanized by CO_2_ inhalation followed by cervical dislocation.

### 2.2 Tumour Inoculations

Lewis‐lung carcinoma (LLC) cells were cultured in high glucose DMEM with 10% fetal bovine serum (FBS) and 1% penicillin/streptomycin. Mice were randomized to experimental groups, then subcutaneously inoculated with 500 000 LLC cells per flank (or PBS for sham). Tumours were allowed to grow for 2, 2.5 or 3.5 weeks (based on preliminary data and previous reports that wasting occurs between 2 and 3 weeks and muscle samples are typically collected between 3 and 4 weeks) [11, 12, 13, 14]. Sham mice were collected at 2.5 weeks. Inoculations were staggered so that collections took place at the same time to avoid batch effects.

### 2.3 EchoMRI

EchoMRIs were performed to assess lean mass and fat mass the day before tumour inoculation and on tissue collection days by members of the University of Ottawa Animal Behaviour Core Facility. Lean mass of tumour‐bearing mice was obtained by subtracting the mass of the tumour (free of skin and excess fat) post‐mortem from the lean mass obtained from EchoMRI.

### 2.4 Single‐Cell RNA Sequencing

Single‐cell suspensions were prepared from whole‐hindlimb muscle samples and were stained for live (Calcein AM) and dead (SYTOX 7AAD) cells. Samples were sent to the Ottawa Hospital Research Institute Flow Cytometry Core to undergo fluorescence‐activated cell sorting (FACS) for live mononuclear cells using the Beckman Coulter MoFlo XDP cell sorter. Cell viability was 91%, 92%, 91% and 93% in sham, 2‐, 2.5‐ and 3.5‐week samples (Figure S4).

scRNAseq was performed on skeletal muscle hindlimbs of sham and 2‐, 2.5‐ and 3.5‐week LLC tumour‐bearing mice by the University of Ottawa Stem Core. Three mice per condition were chosen for this analysis based on similar changes in body mass, lean mass and fat mass throughout the experiment. Muscle samples from all three mice from the same experimental group were pooled, processed for FACS and analyzed simultaneously to avoid batch effects. At least 10 000 freshly sorted live cells were sent to the Ottawa Hospital Research Institute StemCore and prepared for sequencing using the 10× Genomics Chromium System (v3.1) according to manufacturer's instructions, and sequenced on the NextSeq 500 at 10 000 reads per cell. Data were aligned to the mouse genome (mm10) and barcodes, features and matrix files using CellRanger (software and algorithms in Table S5).

### 2.5 Statistical Analyses

Schematics and figures were created using Adobe Illustrator with some images from BioRender. scRNAseq analyses and figures were conducted in R Studio. Differentially expressed genes were calculated using the Wilcoxon rank‐sum statistical test, with *p*_adjusted_ < 0.05 considered statistically significant. All other statistical analyses and figures were performed in GraphPad Prism. One‐way ANOVAs with Tukey's post hoc testing were used to compare four time points, and those with two comparisons used an unpaired *t*‐test with *p* < 0.05 considered statistically significant.

## 3 Results

### 3.1 Body Composition Changes

**FIGURE 1:**
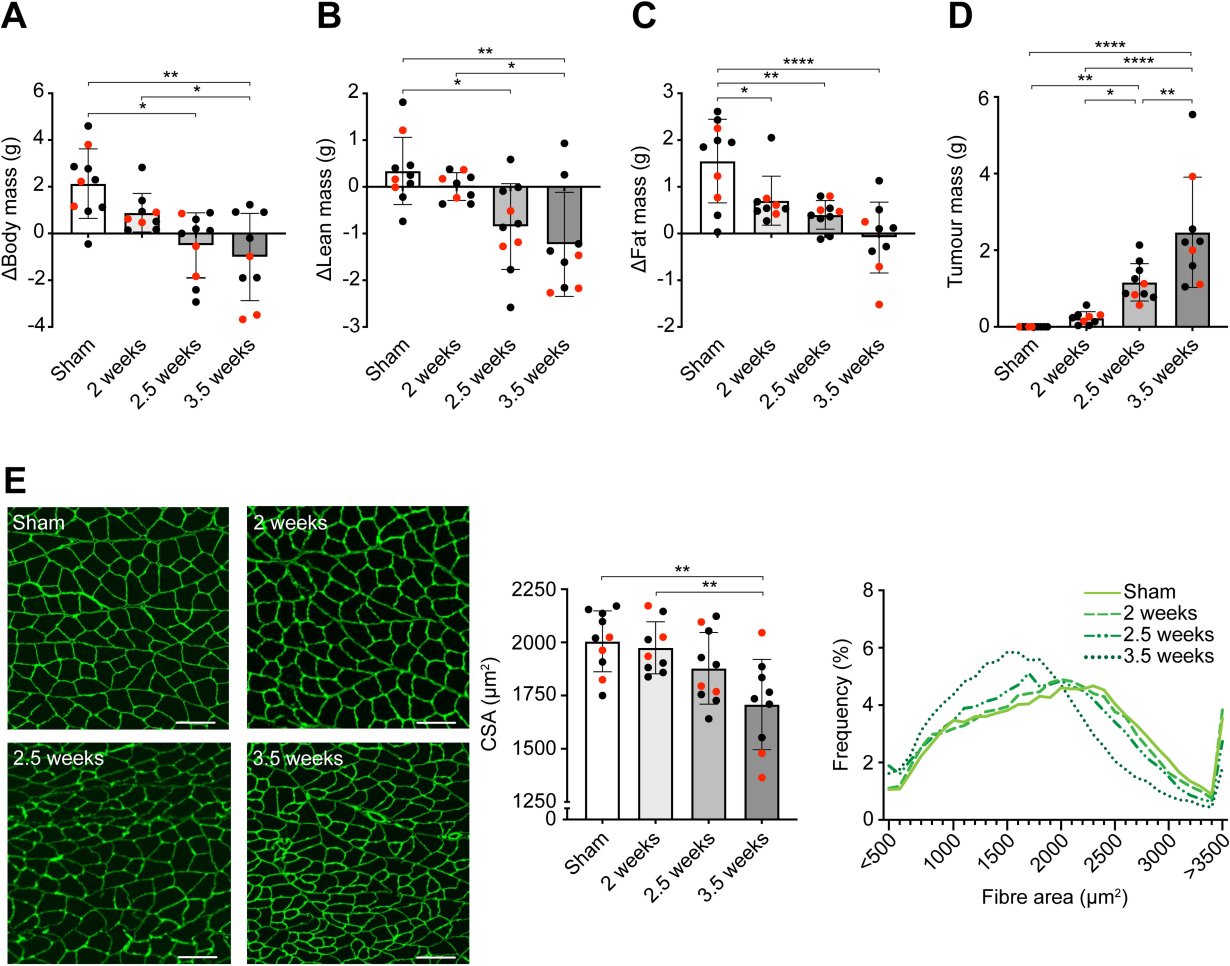
Time course of body composition changes. (A–C) Changes in (A) tumour‐free body mass, (B) tumour‐free lean mass and (C) fat mass from preinoculation to endpoint. (D) Tumour mass. (E) Representative immunofluorescent images of gastrocnemius/soleus complex muscles stained with Laminin and imaged at 20× objective (left panel; where scale bars are 100 μm), muscle cross‐sectional area (middle panel) and frequency distributions of fibre areas per condition (right panel). Data on bar graphs are represented by means ± standard deviation with individual data points, with red data points being those included in the scRNAseq analyses. Data were compared using a one‐way ANOVA, with *p < 0.05, **p < 0.01, ****p < 0.0001.

Body mass changes (Figure 1A) from baseline were positive in sham (+2.13 g) and 2‐week (+0.89 g) tumour‐inoculated conditions and negative at 2.5‐week (−0.51 g) and 3.5‐week (−1.01 g) tumour‐inoculated conditions, where the change in body mass was significantly reduced at 2.5 weeks (*p* = 0.0014) and 3.5 weeks (0.0002) compared to sham and at 3.5 weeks compared to 2 weeks (*p* = 0.0405). Lean mass changes (Figure 1B) from baseline were positive in sham (+0.34 g), similar at 2 weeks (0.0053 g) and negative at 2.5 weeks (−0.85 g) and 3.5 weeks (−1.23 g), where the change in lean mass was significantly reduced at 2.5 weeks (*p* = 0.0134) and 3.5 weeks (*p* = 0.0011) compared to sham and at 3.5 weeks compared to 2 weeks (*p* = 0.0151). Fat mass changes (Figure 1C) from baseline were positive in sham (+1.55 g), 2 weeks (+0.70 g) and 2.5 weeks (+0.40 g) conditions and negative at 3.5 weeks (−0.087 g), where the change in fat mass was significantly reduced at 2 weeks (*p* = 0.0408), 2.5 weeks (*p* = 0.0240) and 3.5 weeks (*p* < 0.0001) compared to sham. Tumour mass (Figure 1D) was significantly increased from sham (0 g) to 2.5 weeks (1.16 g, *p* = 0.0073) and 3.5 weeks (2.47 g, *p* < 0.0001), from 2 weeks (0.23 g) to 2.5 weeks (*p* = 0.0475) and 3.5 weeks (*p* < 0.0001) and from 2.5 to 3.5 weeks (*p* = 0.0030). Myofibre cross‐sectional area (CSA; Figure 1E) was significantly reduced from sham (2004.86 μm) to 3.5 weeks (1707.92 μm, *p* = 0.0022) and from 2 weeks (1974.63 μm) to 3.5 weeks (*p* = 0.0081), with no differences compared to 2.5 weeks (1878.17 μm). These results confirm the presence of wasting in our cohort beginning at 2.5 weeks and increase in severity until 3.5 weeks.

### 3.2 Progressive Changes in Muscle Gene Expression and Cell Populations

**FIGURE 2:**
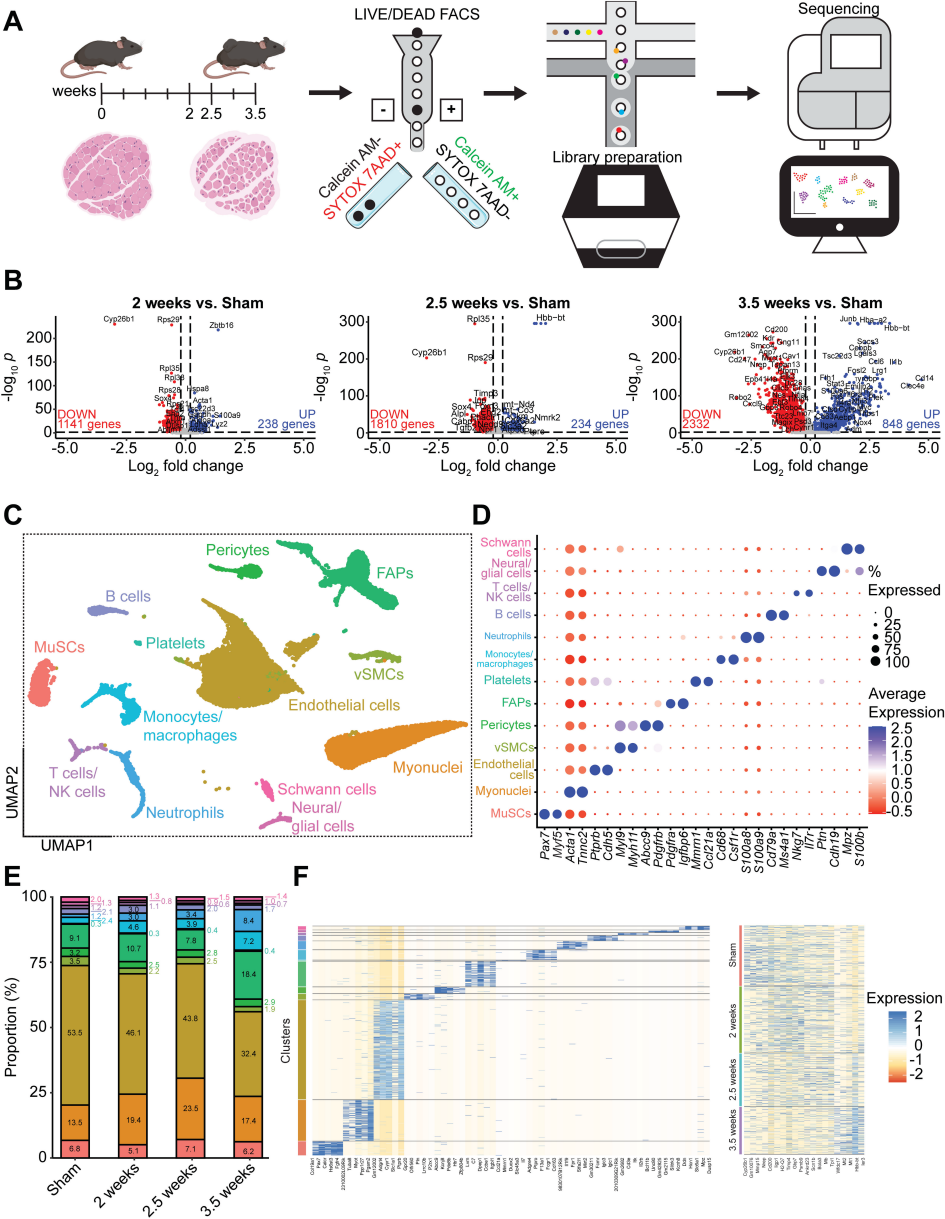
Single‐cell RNA sequencing characterization of time points and cell clusters. (A) Schematic overview of collection time points and preparation for scRNAseq. (B) Volcano plots of differentially expressed genes in all (pooled) cells at 2, 2.5 and 3.5 weeks relative to sham, with significantly upregulated genes (blue) having a log_2_ fold change greater than 0.2 and *p*_adjusted_ < 0.05, and significantly downregulated genes (red) having a log_2_ fold change less than −0.2 and *p*_adjusted_ < 0.05. (C) UMAP plot of cells coloured by clusters. (D) Dotplot of unique markers by cluster. (E) Stacked barplot of cell cluster proportions by condition. (F) Heatmaps of top 5 genes by cluster (left) and by condition (right).

Muscle samples from each time point were sorted for live cells and processed for scRNAseq (Figure 2A). Combined cell populations from a pseudo‐bulk analysis showed a progressive increase in the number of significantly upregulated and downregulated genes at 2 weeks (+238, −1141), 2.5 weeks (+234, −1810) and 3.5 weeks (+848, −2332) compared to sham (Figure 2B). Unsupervised clustering revealed 13 unique cell populations that were annotated using the top differentially expressed genes per cluster (Figure 2C), including MuSCs (*Pax7*/*Myf5*), myonuclei (*Acta1*/*Tnnc2*), endothelial cells (*Ptprb*/*Cdh5*), vascular smooth muscle cells (vSMCs; *Myl9*/*Myh11*), pericytes (*Abcc9*/*Pdgfrb*), FAPs (*Pdgfra*/*Igfbp6*), platelets (*Mmrn1*/*Ccl21a*), monocytes/macrophages (*Cd68*/*Csf1r*), neutrophils (*S100a8*/*S100a9*), B cells (*Cd79a*/*Ms4a1*), T cells/NK cells (*Nkg7*/*Il7r*), neural/glial cells (*Ptn*/*Cdh19*) and Schwann cells (*Mpz*/*S100b*) (Figure 2D). Cell population changes include relative declines in endothelial cells (53.5%–32.4%) and vSMCs (3.5%–1.9%) and increases in FAPs (9.1%–18.4%), monocytes/macrophages (2.4%–7.2%) and neutrophils (1.2%–8.4%) from sham to 3.5 weeks (Figure 2E). Each cell cluster and condition showed transcriptional signatures unique from one another (Figure 2F).

### 3.3 Emergence of a New Cachexia‐Associated MuSC Subcluster

**FIGURE 3:**
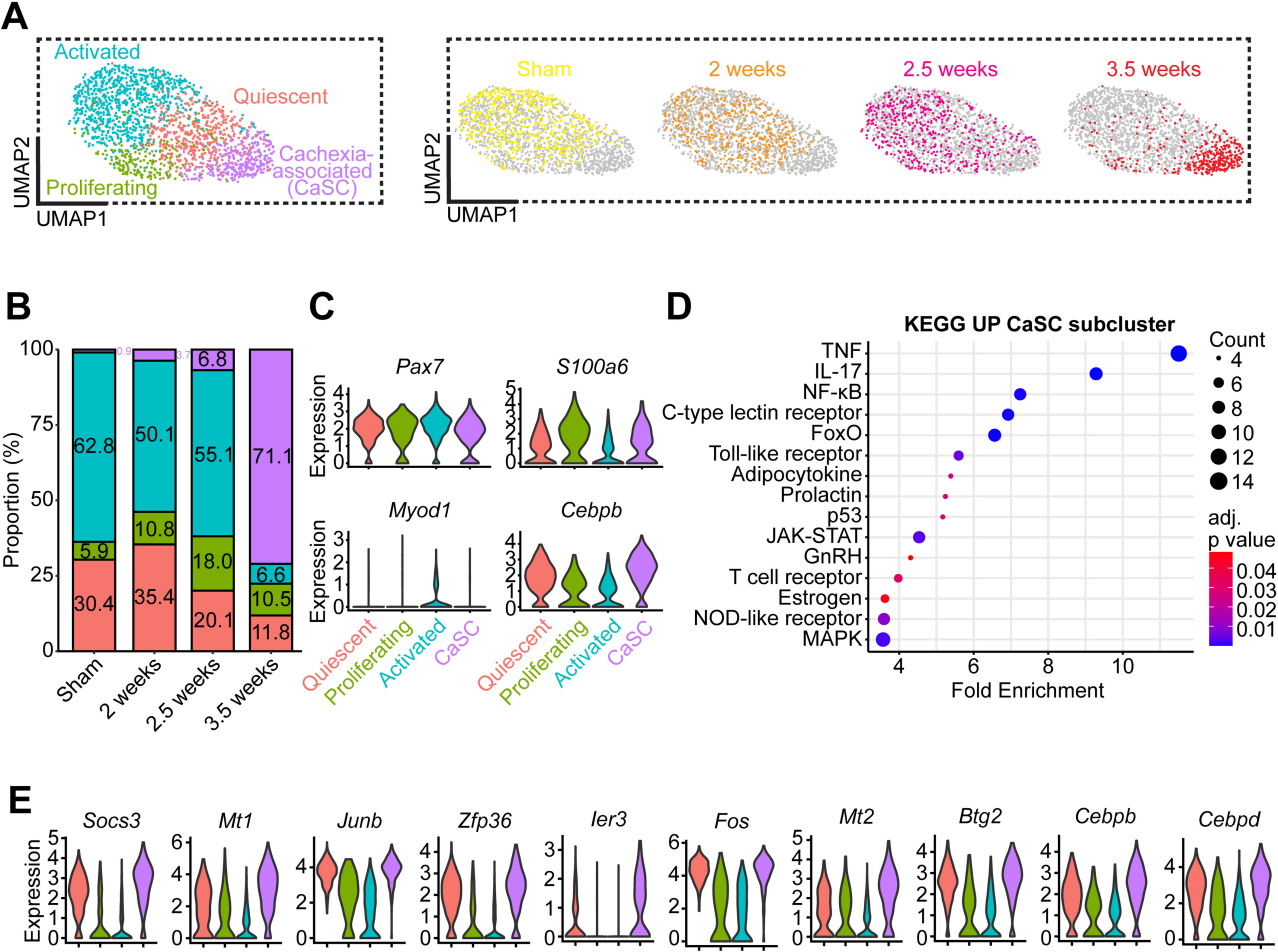
Identification of a new cachexia‐associated subpopulation of MuSCs. (A) UMAP plot of MuSCs coloured by subcluster (left) and highlighted by condition (right). (B) Stacked barplot of MuSCs subcluster proportions by condition. (C) Violin plots of gene expression by subcluster. (D) Significantly enriched KEGG signalling pathways of upregulated genes in CaSC subcluster compared to quiescent, proliferating and activated. (E) Violin plots for the top 10 significantly upregulated genes in the cachexia‐associated satellite cell (CaSC) subcluster compared to all other subclusters.

MuSC subclustering revealed quiescent, proliferating, activated and a novel ‘cachexia‐associated’ (CaSC) subcluster (Figure 3A). The CaSC subcluster proportion drastically increased from sham to 3.5 weeks (0.9%, 3.7%, 6.8%, 71.1%) (Figure 3B). Quiescent (30.4%, 35.4%, 20.1%, 11.8%) and activated (62.8%, 50.1%, 55.1%, 6.6%) proportions decreased from sham to 3.5 weeks, where proliferating proportions increased (5.9%, 10.8%, 18.0%, 10.5%). KEGG pathway analysis on upregulated genes in the CaSC subcluster compared to all other MuSCs revealed significantly enriched signalling related to inflammation (TNF, IL‐17, NF‐κB, C‐type lectin receptor, Toll‐like receptor, NOD‐like receptor, T‐cell receptor, JAK–STAT), stress‐response (p53, FoxO, MAPK) and metabolism/hormonal regulation (adipocytokine, prolactin, GnRH, Oestrogen) (Figure 3D). The top 10 genes upregulated in the CaSC subcluster compared to all other subclusters were *Socs3* (log_2_FC = 2.09; *p*_adjusted_ = 1.12 × 10^−80^), *Mt1* (log_2_FC = 2.49; *p*_adjusted_ = 2.04 × 10^−74^), *Junb* (log_2_FC = 1.34; *p*_adjusted_ = 2.28 × 10^−68^), *Zfp36* (log_2_FC = 1.81; *p*_adjusted_ = 2.48 × 10^−63^), *Ier3* (log_2_FC = 2.65; *p*_adjusted_ = 3.39 × 10^−60^), *Fos* (log_2_FC = 1.29; *p*_adjusted_ = 1.21 × 10^−57^), *Mt2* (log_2_FC = 2.22; *p*_adjusted_ = 3.60 × 10^−55^), *Btg2* (log_2_FC = 1.41; *p*_adjusted_ = 5.48 × 10^−53^), *Cebpb* (log_2_FC = 1.65; *p*_adjusted_ = 8.23 × 10^−52^) and *Cebpd* (log_2_FC = 1.54; *p*_adjusted_ = 6.41 × 10^−47^) (Figure 3E). The newly characterized CaSC subcluster reveals gene expression that has been described in several previous studies to cause MuSC dysfunction in cancer cachexia [4, 15, 16, 17, 18, 19].

### 3.4 MuSCs From 3.5‐Week Tumour‐Bearing Mice Show Activation Phenotype

**FIGURE 4:**
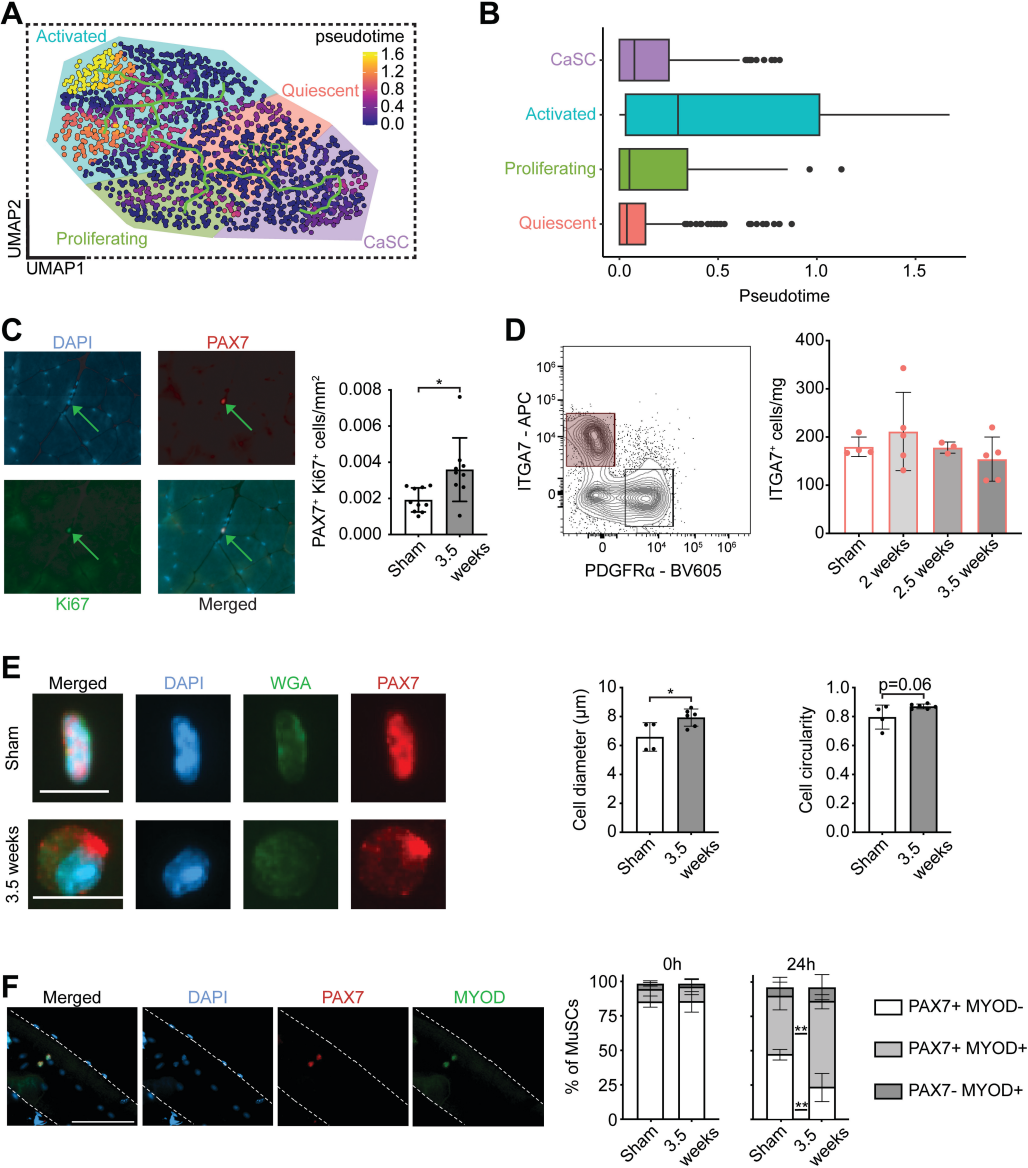
MuSCs from 3.5‐week tumour‐bearing muscle show characteristics of activation. (A) UMAP plot of MuSCs coloured by pseudotime trajectory analysis, with the green line indicating branching starting from Quiescent MuSCs and (B) boxplot of pseudotime values split by subcluster. (C) Representative immunofluorescent images of PAX7/Ki67/DAPI staining on muscle cross‐sections, imaged at 20× objective (left) and PAX7^+^Ki67^+^ counts (right) compared using an unpaired *t*‐test. (D) Representative flow cytometry plot highlighting ITGA7^+^ cells (left; gated on CD31^−^CD45^−^ cells and are PDGFRα^−^) and ITGA7^+^ counts (right) compared using a one‐way ANOVA. (E) Representative immunofluorescent images of MuSCs isolated from sham and 3.5‐week tumour‐bearing muscle via MACs, cultured for 30 min, fixed and stained with PAX7, WGA and DAPI and imaged at 40× objective (where scale bars are 10 μm), with cell diameter and cell circularity compared using unpaired *t*‐tests. (F) Representative immunofluorescent images of PAX7^+^MYOD^+^ cells on an individual EDL myofibre, imaged at 20× objective (where scale bar is 100 μm) and stacked barplots of the proportions of PAX7^+^MYOD^−^, PAX7^+^MYOD^+^, PAX7^−^MYOD^+^ cells immediately after isolation (0 h; *n*_sham_ = 3, *n*_3.5‐weeks_ = 5) and after 24 h (*n*_sham_ = 4, *n*_3.5‐weeks_ = 7) in culture and compared using an unpaired *t*‐test. PAX7^+^Ki67^+^ counts, ITGA7^+^ counts, cell diameter, cell circularity and PAX7/MYOD proportions are represented by means ± standard deviation with individual data points where applicable. *p < 0.05, **p < 0.01.

Pseudotime branch analysis shows branching from quiescent MuSCs to the CaSC subcluster, away from proliferating and activated subclusters (Figure 4A). As CaSCs had higher pseudotime values than quiescent, indicating further transcriptional distance from a stem‐like state (Figure 4B), and several top differentially expressed genes are involved in cell activation (*Junb*, *Fos*), we compared the activation potential of MuSCs from cachectic muscle to sham. Despite an increase in proliferating (PAX7^+^Ki67^+^) cells at 3.5 weeks compared to sham (Figure 4C; 1.87‐fold; *p* = 0.0122), no changes in ITGA7^+^ cell numbers were observed via flow cytometry (Figure 4D; *p* = 0.4271). Consistent with a more activated profile, MuSCs isolated from 3.5‐week tumour‐bearing muscle were larger (1.20‐fold; *p* = 0.0266) and tended to be more circular (1.09‐fold; *p* = 0.0614) compared to those isolated from sham (Figure 4E). Additionally, MuSCs had similar proportions of PAX7^+^MYOD^−^ (*p* = 0.9746), PAX7^+^MYOD^+^ (*p* = 0.7112) and PAX7‐MYOD+ (*p* = 0.3690) cells on individual myofibres isolated from sham and 3.5‐week tumour‐bearing mice; however, when cultured for 24 h, MuSCs from 3.5‐week tumour‐bearing muscle had less PAX7^+^MYOD^−^ (0.51‐fold; *p* = 0.0040) and more PAX7^+^MYOD^+^ (1.48‐fold; *p* = 0.0226) cells (Figure 4F). These results demonstrate that CaSCs have features of activation.

### 3.5 Characterization of CaSC Subcluster

**FIGURE 5:**
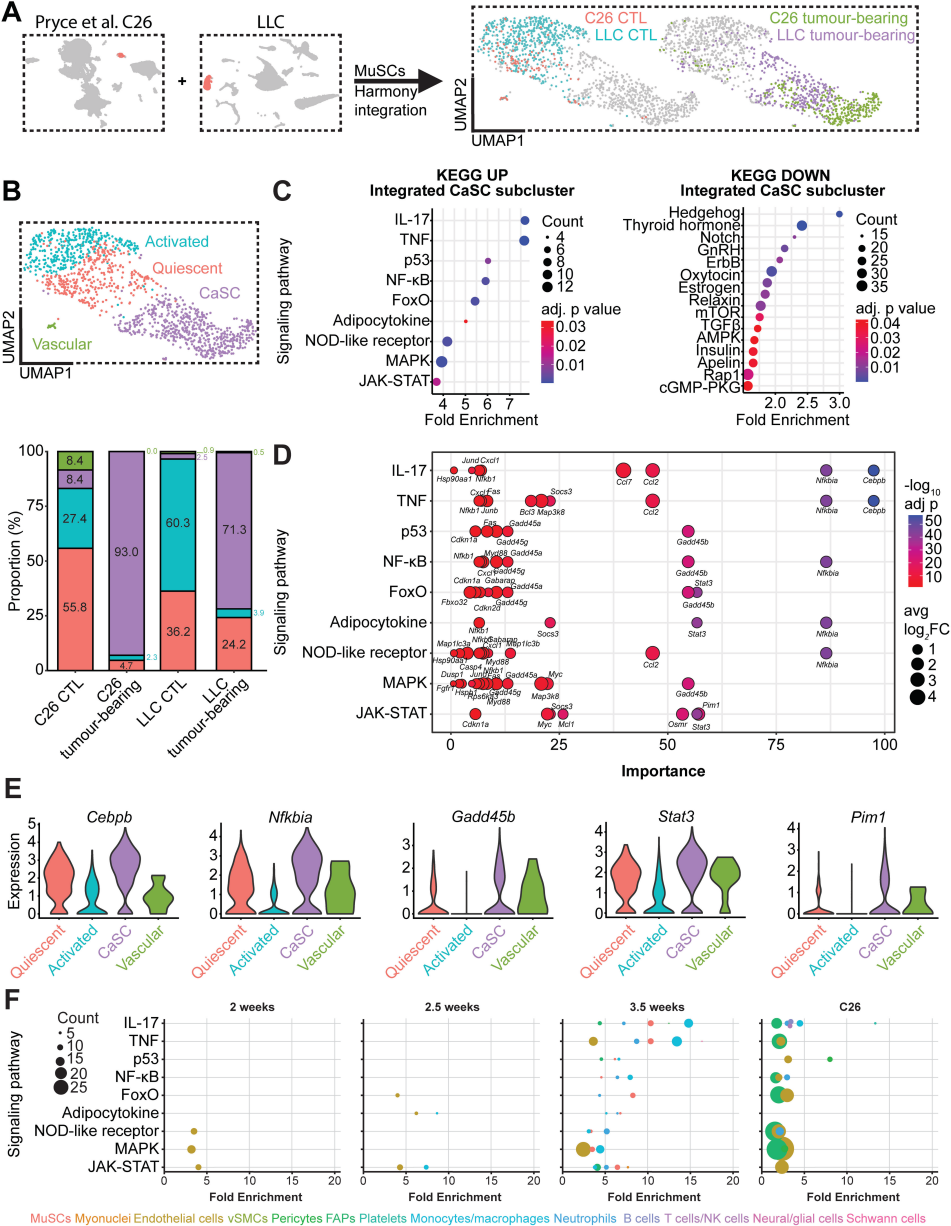
Characterization of the CaSC subcluster. (A) Data integration of MuSCs from publicly available C26 dataset [6] with sham and 3.5‐week MuSCs from our LLC data using the Harmony package in R. UMAP plot of MuSCs coloured by condition. (B) UMAP plot of integrated MuSCs coloured by subcluster (top) and stacked barplot of integrated MuSCs subcluster proportions by condition (bottom). (C) Significantly enriched upregulated (left) and downregulated (right) KEGG signalling pathways in integrated CaSC. (D) Dotplot of gene importance to upregulated signalling pathways in integrated CaSC. Significant genes (defined by log_2_ fold change > 0.2 and *p*_adjusted_ < 0.05 in integrated CaSCs compared to all others) in each pathway were scored based on importance, which was calculated by multiplying ‐log_10_ adjusted *p* value with the average log_2_ fold change for each gene. (E) Violin plots of gene expression split by subcluster of the genes highlighted to be the most important drivers of CaSC gene expression. (F) Dotplot of enriched KEGG signalling pathways in different cell types within the muscle, with selected pathways being those identified as upregulated in the integrated CaSC subcluster. Dots are coloured by cluster, dot size is the number of genes that are upregulated in that pathway, and plots are split by condition (2, 2.5 and 3.5 weeks compared to sham in LLC model, C26 tumour‐bearing compared to sham in C26 model).

To further characterize the CaSC subcluster, we integrated MuSCs (sham and 3.5 weeks) with a scRNAseq dataset of C26‐tumour‐bearing mice [6] using harmony (Figure 5A). After integration, MuSCs were reprocessed and clustered to quiescent, activated, vascular and CaSC cells. Like what was observed in the LLC model, MuSCs from C26 tumour‐bearing mice shift in subcluster proportions towards the CaSC subcluster (8.4%–93%) (Figure 5B). We confirmed that the gene expression signature from the CaSC subcluster is exclusive to cachexia by scoring cells based on upregulated genes in the integrated subcluster and compared it to MuSCs from aging [20], denervation [21], irradiation [9], muscular dystrophy [22] and regeneration [10] (Figure S8). KEGG pathway analysis on differentially expressed genes in the integrated CaSCs found significantly enriched signalling related to inflammation (IL‐17, TNF, NF‐κB, NOD‐like receptor, JAK–STAT), stress response (p53, FoxO, MAPK) and metabolism/hormonal regulation (adipocytokine) (Figure 5C), all of which were upregulated in the CaSC subcluster in LLC tumour‐bearing MuSCs. Additionally, enriched downregulated signalling related to cell fate determination (hedgehog, notch, TGFβ), endocrine signalling (thyroid hormone, gnrh, oxytocin, oestrogen, relaxin, insulin, apelin), metabolism (mTOR, AMPK) and growth factor and secondary messengers (ErbB, Rap1, cGMP‐PKG) was observed. To determine the main drivers of the upregulated signalling pathways, we identified pathway‐specific genes that were upregulated in the CaSC subcluster and determined their ‘importance’ as ‐log_10_ adjusted *p* value multiplied by the average log_2_ fold change (Figure 5D). *Cebpb* (1.71‐fold; *p*_adj_ = 1.10 × 10^−57^), *Nfkbia* (1.94‐fold; *p*_adj_ = 2.92 × 10^−45^), *Gadd45b* (2.21‐fold; *p*_adj_ = 1.90 × 10^−25^), *Stat3* (1.39‐fold; *p*_adj_ = 1.51 × 10^−41^) and *Pim1* (2.50‐fold; *p*_adj_ = 1.42 × 10^−23^) were identified as the most important drivers for the CaSC subcluster's gene expression changes and were upregulated compared to in other subclusters (Figure 5E). We next examined signalling pathways that were upregulated in CaSCs in other cell types, at all timepoints, compared to sham, and in C26 tumour‐bearing mice compared to their respective controls (Figure 5F). There was a gradual increase in several signalling pathways from the CaSC gene signature in various cells until 3.5 weeks and in C26 tumour‐bearing muscle, with signalling mainly observed in MuSCs, endothelial cells, FAPs, monocytes/macrophages and neutrophils, revealing that this CaSC gene signature is, in part, reflected in other cell populations.

### 3.6 Increases in Immune Cell Populations Precede Wasting

**FIGURE 6:**
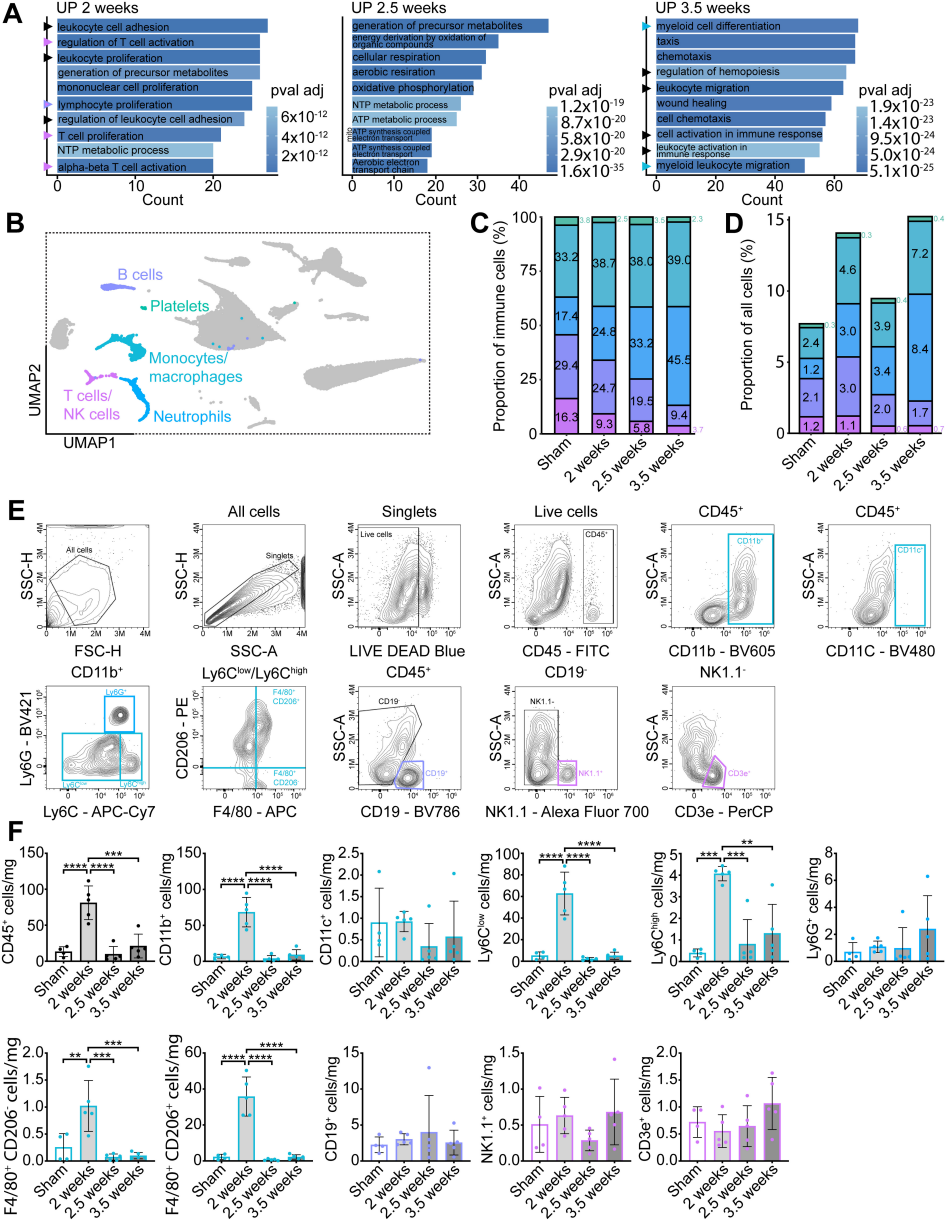
Timing of immune cell changes with cancer cachexia. (A) Top 10 upregulated GO terms in all cells at 2, 2.5 and 3.5 weeks relative to sham. (B) UMAP plot highlighting immune cell populations. (C–D) Stacked barplots of immune cell proportions relative to (C) other immune cells and (D) all cells by condition. (E) Representative plots and gating strategy for muscle immune cells, including myeloid populations [All cells ➔ Singlets ➔ Live cells (LIVE DEAD Blue^−^) ➔ CD45^+^ ➔ CD11b^+^, CD11c^+^ ➔ (from CD11b^+^) Ly6C vs. Ly6G ➔ (from Ly6C^low/high^) F4/80 vs. CD206] and lymphocyte populations [CD45^+^ ➔ CD19^+^ ➔ (from CD19^−^) NK1.1^+^ ➔ (from NK1.1^−^) CD3e^+^], where the title indicates the cells that were previously gated on, and x and y axes indicate the channels used for gating. (F) Immune cell counts per muscle weight, compared using a one‐way ANOVA with **p < 0.01, ***p < 0.001, ****p < 0.0001.

As a large portion of enriched signalling in CaSCs was immune‐related, we next examined changes to skeletal muscle immune cells throughout cancer cachexia. Gene Ontology (GO) Biological Processes analyses were performed on significantly upregulated genes in pooled cells using the org. Mm.eg.db package to observe changes in overall gene expression patterns in the muscle. Of the top GO terms, 7/10 at 2 weeks were immune related, which were absent at 2.5 weeks (0/10) and reappeared at 3.5 weeks (6/10), demonstrating an overall increase in immune‐related signalling in the muscle with cancer (Figure 6A). Immune cells encompassed 5/13 of our observed clusters (Figure 6B) and their proportions shifted towards myeloid populations (neutrophils and monocytes/macrophages) and away from lymphocyte populations (B‐cells and T‐cells/NK‐cells) from sham to 3.5 weeks (Figure 6C). When immune cell proportions were quantified relative to all cells, there was an increase from sham (7.2%) to 2 weeks (12.0%), which dropped at 2.5 weeks (10.3%) and increased again at 3.5 weeks (17.7%) (Figure 6D). Flow cytometry confirmed these early increases in immune cells. Total CD45+ immune cells (6.1‐fold, *p* < 0.0001) and other myeloid cells such as CD11b^+^ (10.3‐fold, *p* < 0.0001), Ly6C^low^ (11.8‐fold, *p* < 0.0001) and Ly6C^high^ (10.2‐fold, *p* = 0.0002) monocytes, F4/80^+^CD206^−^ (4.0‐fold, *p* = 0.0039) pro‐inflammatory and F4/80^+^CD206^+^ (15.7‐fold, *p* < 0.0001) anti‐inflammatory macrophages increased at 2 weeks compared to sham, which returned to baseline by 2.5 weeks and remained low at 3.5 weeks (Figure 6F). No changes to CD11c^+^ myeloid cells (*p* = 0.4532), Ly6G^+^ neutrophils (*p* = 0.3549), CD19^+^ B‐cells (*p* = 0.7939), NK1.1^+^ natural‐killer cells (*p* = 0.2729) or CD3e+ T‐cells (*p* = 0.1949) cells were observed. Flow cytometry in the spleen revealed significant increases in myeloid populations (total, CD11b^+^, CD11c^+^, Ly6C^low^, Ly6C^high^, Ly6G^+^, F4/80^+^CD206^−^, F4/80^+^CD206^+^) at 3.5 weeks compared to sham, 2 and 2.5 weeks (*p* < 0.0001), an increase in CD3e^+^ cells (*p* < 0.01) and a decrease in NK1.1^+^ cells (*p* < 0.05) at 2.5 and 3.5 weeks relative to sham and 2 weeks (Figure S9). Complete blood cell counts revealed an increase in blood neutrophils (*p* < 0.0001) and a decrease in lymphocytes (*p* < 0.0001) at 3.5 weeks compared to sham, 2 weeks and 2.5 weeks (Table S7). No significant changes to immune cell content were observed in the tumour (Figure S10). These findings demonstrate a transient increase in myeloid cell populations in the muscle at 2 weeks that precede cachexia, which are partially reflected in the spleen and blood.

### 3.7 Changes in Endothelial Cells and FAPs Precedes Wasting

**FIGURE 7:**
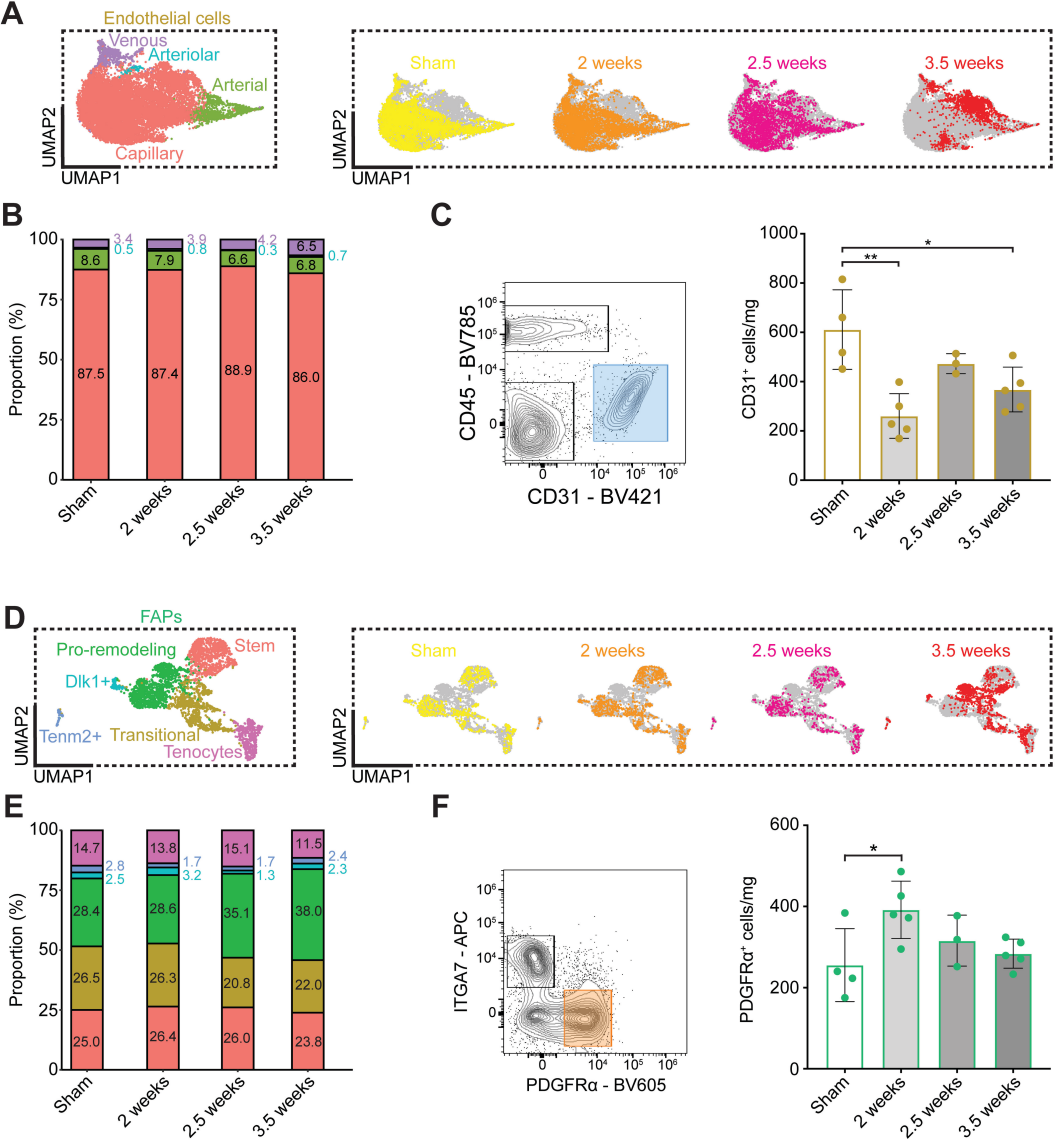
Changes to endothelial cells and FAPs with cancer cachexia. (A) UMAP plots of endothelial cells coloured by subcluster (left) and highlighted by condition (right). (B) Stacked barplot of endothelial cell subcluster proportions by condition. (C) Representative flow cytometry plot highlighting CD31^+^ cells (left; gated on Live cells and are CD45^−^) and CD31^+^ counts (right). (D) UMAP plots of FAPs coloured by subcluster (left) and highlighted by condition (right). (E) Stacked barplot of FAP subcluster proportions by condition. (F) Representative flow cytometry plot highlighting PDGFRα^+^ cells (left; gated on CD31^−^CD45^−^ cells and are ITGA7^−^) and PDGFRα^+^ counts (right). Flow cytometry was compared using a one‐way ANOVA with *p < 0.05, **p < 0.01.

Endothelial cells [23, 24] and FAPs [25, 26] are also key regulators of MuSCs. Endothelial cell subclustering revealed capillary, arterial, arteriolar and venous subclusters using previously‐described markers [9, 22] and demonstrated a gradual change in gene expression signature from sham to 3.5 weeks (Figure 7A). Capillary (87.5%, 87.4%, 88.9% and 86.0%), arterial (8.6%, 7.9%, 6.6% and 6.8%), arteriolar (0.5%, 0.8%, 0.3% and 0.7%) and venous (3.4%, 3.9%, 4.2% and 6.5%) cell proportions remained relatively stable from sham to 3.5 weeks (Figure 7B) despite lower overall endothelial cell proportions. Flow cytometry revealed less CD31+ cells at 2 weeks (0.43‐fold; *p* = 0.0014) and 3.5 weeks (0.60‐fold; *p* = 0.0213) compared to sham (Figure 7C). FAPs subclustering revealed stem, transitional, pro‐remodelling, Dlk1+, Tenm2+ and tenocyte subpopulations based on previously published datasets [9, 10, 27] and demonstrated a gradual change in gene expression signature from sham to 3.5 weeks (Figure 7D). The ‘transitional’ subcluster had a unique gene signature to previous literature, and pseudotime branch analysis revealed a path through the transitional subcluster from stem to pro‐remodelling in healthy FAPs (Figure S11). There was a relative decrease in transitional (26.5%, 26.3%, 20.8% and 22.0%) and increase in pro‐remodelling (28.4%, 28.6%, 35.1% and 38.0%) subclusters from sham to 3.5 weeks (Figure 7E). Flow cytometry revealed a transient increase in PDGFRα+ cells at 2 weeks compared to sham (1.53‐fold; *p* = 0.0398) but was not different at 2.5 weeks (*p* = 0.6439) or 3.5 weeks (*p* = 0.9201). These results demonstrate a decrease in endothelial cell content at 2 and 3.5 weeks, where FAPs transiently increased at 2 weeks and returned to baseline by 2.5 weeks. Changes in endothelial cell and FAP content, along with immune cells, reveals a change to the muscle microenvironment that precedes muscle wasting with cancer cachexia.

### 3.8 CaSCs Have Decreased Gene Expression for Cell Matrix Adhesion

**FIGURE 8:**
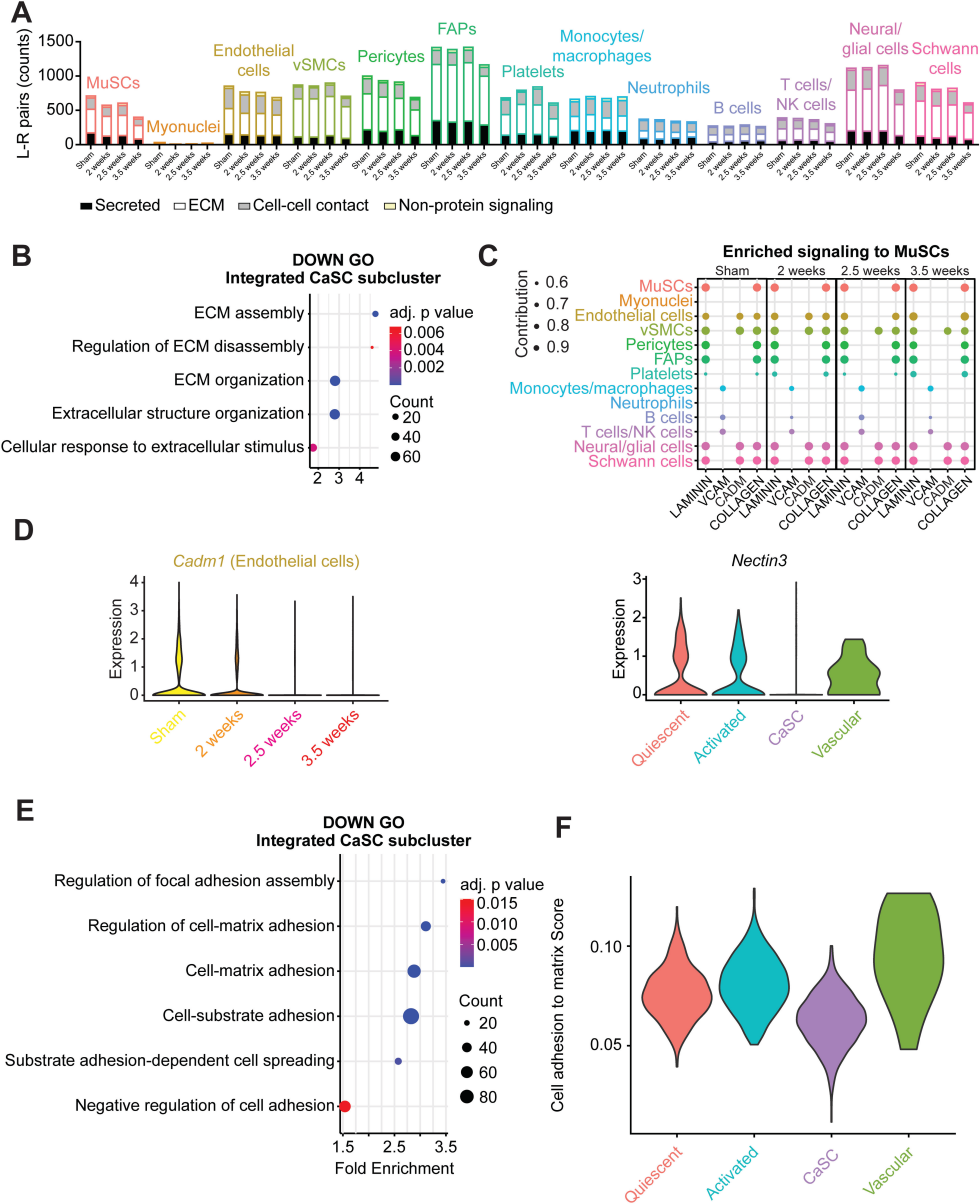
Decreased signalling related to cell‐matrix interactions in satellite cells from cachectic muscle. (A) Stacked barplot of ligand‐receptor pairs for each cell cluster by condition, with bars coloured by signalling type (secreted = black, ECM = white, cell–cell contact = grey, nonprotein signalling = tan). (B) GO terms of downregulated genes in integrated CaSC relative to other subclusters related to the ECM. (C) Dotplot of enriched ECM signalling pathways received by MuSCs for at each time point. (D) Violin plot for *Cadm1* expression in endothelial cells by cluster, and *Nectin3* expression in integrated MuSCs and split by subcluster. (E) GO terms of downregulated genes in CaSCs relative to other subclusters related to cell‐matrix interactions. (F) Violin plot of cell scoring for genes involved in cell adhesion to the matrix.

Given that the muscle microenvironment is an important regulator of MuSC function, we used CellChat [8] analyses to identify communication patterns between cell types within the muscle microenvironment with cancer cachexia. There was an overall decrease in cell communication (identified by enriched ligand‐receptor pairs) from sham to 3.5 weeks in all cells except monocytes/macrophages (Figure 8A). GO analyses in CaSCs revealed a downregulation of genes related to ECM organization and response (Figure 8B). We next assessed communication patterns received by MuSCs and focused on ECM‐related signalling. Laminin, VCAM, CADM and collagen were enriched ECM‐related signalling received by MuSCs (Figure 8C). Of the enriched signals to the MuSCs, the most obvious change was the loss of CADM signalling from endothelial cell s at 2.5 and 3.5 weeks. *Cadm1* (ligand) expression was significantly reduced in endothelial cell s at 2 weeks (−0.39‐fold; *p*_adjusted_ = 0.00217), 2.5 weeks (−0.63‐fold; *p*_adjusted_ = 1.80 × 10^−13^) and 3.5 weeks (−1.37‐fold; *p*_adjusted_ = 1.25 × 10^−38^) compared to sham, and *Nectin3* (receptor) signalling, although not significantly different, is mostly absent in the CaSC subcluster (Figure 8D). As the CADM pathway plays a pivotal role in MuSC adhesion [28], we looked for indicators of cell‐matrix interactions and found enriched GO terms in downregulated genes related to cell‐matrix adhesion (Figure 8E). We created another gene score using msigdbr and used a gene list from a Gene Ontology dataset (GOBP_CELL_MATRIX_ADHESION), where a reduction in CaSCs (−25.45%) was found compared to other subclusters. These results indicate that CaSCs have decreased ECM‐related signalling, which helps explain impaired MuSC differentiation with cancer cachexia.

## 4 Discussion

The goal of this study was to comprehensively measure changes to the skeletal muscle microenvironment throughout cancer cachexia. While other studies have reported changes to specific components of the microenvironment, none have measured all components within the microenvironment and when these changes occur. Our discovery of a novel CaSC subcluster and that several cell type changes precede muscle wasting are crucial to understanding mechanisms of muscle wasting with cancer cachexia.

We describe a new subpopulation of ‘cachexia‐associated’ MuSCs, which contains a gene signature characterized by inflammatory [4, 15, 16, 17] and cell stress [18, 19] signalling pathways. The CaSC subpopulation was also present in C26 tumour‐bearing mice, was exclusive to cancer cachexia and contained several gene expression similarities to MuSCs from human PDAC patients (Figure S8). Each pathway upregulated in CaSCs has been previously described to independently cause MuSC dysfunction and/or muscle atrophy. Additionally, impaired muscle regeneration in cancer cachexia has been well‐characterized [4, 7, 29]. We report that CaSCs comprise 71.1% of MuSCs at 3.5 weeks in LLC tumour‐bearing mice and 93.0% in C26 tumour‐bearing mice. Previous evidence of impaired MuSC contributions to regeneration includes 74.1% less embryonic myosin heavy chain‐positive fibres in C26 tumour‐bearing muscle [4], ~50% reduction in fibre size after injury in LLC tumour‐bearing muscle [29] and reduced differentiation (~35%) and fusion (~50%) of MuSCs from cachectic muscle when differentiated ex vivo [7], which confirms impaired MuSC differentiation in C26 and LLC models. Our results suggest that impaired MuSC differentiation in cachexia may be due to the CaSC gene signature present in the majority of MuSCs in cachectic muscle.

MuSCs contribute to myofibre size maintenance in the absence of regeneration. Knockdown of MuSC‐specific C/EBPβ (upregulated in MuSCs and tumours to promote cachexia) [7, 29, 30] led to greater myofibre size in vivo [31], demonstrating that changes in MuSC‐specific gene expression are sufficient to alter myofibre size. The CaSC subcluster has high *Cebpb*, *Stat3*, *Socs3* and *Nfkbia* expression, which have all been shown to independently induce myotube atrophy [4, 7, 16, 17, 29, 30]. CaSCs display key hallmarks of transcriptional signatures characterized in cancer cachexia, and although many of these signatures have been described previously, the novelty of our findings comes from identifying the emergence of a unique population, and that it occurs following several changes to the muscle microenvironment during cancer cachexia. While we do not directly show that these cells are responsible for muscle atrophy in vivo, there is sufficient evidence that these gene expression signatures in CaSCs lead to impaired MuSCs differentiation, which can subsequently contribute to atrophy.

MuSCs in cachectic muscle show features of activation as evidenced by increased Ki67 and MYOD protein staining. While there is evidence of muscle damage in cancer cachexia [4, 5, 32], we propose that CaSCs are exhibiting a stress response and not a true repair mechanism. While CaSCs display some features of activated MuSCs, their transcriptional signature shows a distinct branching from activated MuSCs with pseudotime and is likely a result of systemic inflammation and altered intramuscular signalling causing this altered cell state. We compared upregulated genes in CaSCs to activated MuSCs in healthy mice [10] and found a ~ 21% overlap in gene expression, many of which were stress and inflammation related, including GO terms for the regulation of cytokine production, cellular response to IL‐6, NF‐kappaB signal transduction, cellular response to chemokine and the regulation of myeloid cell differentiation (data not shown). Therefore, although CaSCs demonstrate features of activation, they are unique from activated MuSCs in healthy muscle. As part of the response to inflammatory cues, several important MuSC regulators, including *Cebpb* and *Nfkbia*, are overexpressed. These MuSC regulators have been shown to impair differentiation and protect against apoptosis [4, 29].

*Cebpb* and *Nfkbia* are known regulators of senescence‐associated secretory phenotype [33] and correspond with enriched p53 signalling in CaSCs. While these factors indicate senescence as a potential mechanism of dysfunction, these cells do not appear to be senescent. Senescence‐associated gene scoring revealed no difference between MuSC subclusters, and C2C12 myoblasts treated with LLC conditioned media showed similar SA‐β‐galactosidase staining as controls (Figure S12). Additionally, MuSCs in 3.5‐week tumour‐bearing muscle tend to have more PAX7^+^EdU^+^ cells following cardiotoxin injury (Figure S13), which indicates that the mechanism of dysfunction is something other than senescence. While the source of the signals that cause MuSC dysfunction is not well‐known, it is likely due to the combined effects of tumour‐secreted factors [30], systemic inflammation [34] and local changes within the muscle, interacting with these signalling pathways to cause MuSC dysfunction in cancer cachexia.

As previously reported, tumour‐bearing mice show abundant immune‐related gene expression in skeletal muscle [6, 12]. Systemic inflammation is a hallmark of cancer cachexia [34] and is reflected in the spleen and blood at 3.5 weeks in the present study by increases in myeloid cell populations. While others report muscle inflammation at endpoint [6, 35, 36], we observed that total immune and myeloid cell populations increased at 2 weeks, returned to baseline by 2.5 weeks and remained diminished at 3.5 weeks indicating that immune cell accumulation precedes wasting. Furthermore, our data support previous reports that cancer cachexia is associated with increased myeloid cell proportions in the muscle [6, 36] and expands on these findings by demonstrating a transient increase in absolute immune cell content, which provides a more physiological view of the inflammatory environment in skeletal muscle. The increase in immune cell proportion at 2 weeks and subsequent decline at 2.5 weeks is reflected in our scRNAseq proportion analysis; however, the second increase in immune cell proportions at 3.5 weeks may be explained by the relative decrease in other abundant populations such as endothelial cells or by differences normalizing by muscle weight. An early, transient increase in immune cells was observed in a melanoma model of cachexia, increasing at 1‐week and returning to baseline by 3 weeks [24]. Although mechanisms of immune cell homing to the muscle are not well understood, several components of the muscle microenvironment, including MuSCs, FAPs and endothelial cells, communicate with nonmuscle‐resident immune cells and could influence immune cell infiltration [10, 37].

Some of the most downregulated GO terms in cachectic muscle were related to regulation of the microvasculature, which is essential for gas exchange, nutrient supply and cell signalling [23]. Capillary content is important for regulating components of the muscle microenvironment and is lower in cancer patients [23, 24]. In the present study, both endothelial cell proportion and absolute cell content decreased at 2 weeks, preceding muscle atrophy, which aligns with a recent study that reported decreased vascular density that preceded muscle atrophy in mouse models of cancer and that restoring endothelial cell function preserved muscle mass [24]. Together, along with their role in regulating MuSC function [23] and the immune/inflammatory response [24], suggest that endothelial cell dysfunction is involved in initiating muscle wasting with cancer cachexia. Indeed, the loss of CADM signalling from endothelial cells to MuSCs was apparent at 2.5 weeks and may help to explain MuSC dysfunction with cancer cachexia.

While overall cell communication in the muscle went down with cachexia, the most enriched signals received by MuSCs at 3.5 weeks are ECM‐related. ECM is essential for maintaining muscle integrity, force transduction and for providing cell signalling cues [38]. MuSCs are regulated in the niche by the ECM through ECM composition and density, fibril arrangement, stiffness and cell adherence to the matrix [38]. Our findings in CaSCs demonstrated decreased gene expression related to cell adhesion to the matrix and specifically highlight that signals for the CADM signalling pathway are reduced to the MuSCs. M‐cadherin and other cadherin proteins are essential for MuSC‐myotube fusion, and loss of M‐cadherin impairs differentiation [28]; therefore, suggesting that differentiation defects reported in cancer cachexia [4, 15, 17, 19, 30] may result from impaired cell–cell and cell‐matrix adhesion. Future work should focus on preventing early endothelial cell declines and immune cell infiltration during cancer cachexia to mitigate MuSC dysfunction and muscle atrophy.

## 5 Conclusions

We observed changes to several components of the muscle microenvironment, such as a decline in endothelial cells and a transient increase in FAPs and immune cells, that precedes muscle wasting. A combination of changes in the muscle microenvironment and external cues contributes to a unique cachexia‐associated subpopulation of MuSCs. The changes to the muscle microenvironment and when they occur provide valuable insight into muscle wasting during cancer.

## 6 Limitations

Our findings are primarily limited to male mice as they exhibit more severe cachexia than females [11, 13]; however, our findings in males provide a basis for further targeted investigation in females. It is important to discern the scale difference between cell proportions (from scRNAseq) and absolute counts (from flow cytometry) changes throughout our study. Although most of the observed trends were similar between analyses, scRNAseq proportion analyses can overrepresent abundant populations due to limited cell capture, underrepresent fragile cells due to higher processing time and harsher digestion and remove ‘stressed’ cells throughout standard scRNAseq pipelines. Therefore, flow cytometry is a better measure of cell changes. Additionally, there is a large portion of ‘activated’ MuSCs in our dataset (including in sham), which is likely a result of early activation due to FACS prior to scRNAseq [39]. After integrating C26 MuSCs with our dataset, subclustering analyses revealed slightly different subclusters compared to LLC MuSCs alone, which can be attributed to the presence of the vascular cluster in the C26 model. Additionally, compiling all cells for GO term and differential gene expression analyses is biased by the number of each cell type that comprises the total cell population; as this approach would potentially skew data if there are drastic changes in cell populations. Finally, as we do not directly show that CaSCs cause muscle wasting, future work needs to be conducted to link these cells to muscle atrophy with cachexia.

## Funding

Authors were supported by an Ontario Graduate Scholarship (A.B.); a Chilean National Agency for Research and Development Scholarship, a uOttawa/CHEO Research Institute‘s Doctoral Fellowship for Advancement of Biological Perspectives for Exercise Interventions Across the Lifespan and a uOttawa Éric Poulin Centre for Neuromuscular Disease Scholarship in Translational Research and a uOttawa Brain and Mind Research Institute (N.C.); a Queen Elizabeth II Graduate Scholarship in Science and Technology (A.S.); and a Canadian Institute of Health Research (CIHR) Master‘s Graduate Scholarship (N.S.). Funding was provided by the CIHR Project Grant (#195826) awarded to M.D. and N.W.B.

## Ethics Statement

This manuscript complies with the ethical guidelines for authorship and publishing in the Journal of Cachexia, Sarcopenia and Muscle.

## Conflicts of Interest

The authors declare no conflicts of interest.

## Supporting information


**Figure S1:** Time course of body composition changes in female mice with LLC model of cancer cachexia. Changes in (A) Tumour‐free body mass, (B) tumour‐free lean mass and (C) fat mass. (D) Tumour mass. (E) Representative images of muscle cross sections stained for laminin and taken at 20× objective (left panel; scale bar is 100 μm). Average cross‐sectional area of muscle fibres from gastrocnemius/soleus complex (middle panel). Muscle fibre size distribution (right panel). Data are represented as means ± standard deviation with individual data points and were compared using a one‐way ANOVA (**p < 0.01).

**Figure S2:** Food consumption and grip strength over the course of cancer cachexia in male mice. (A) Weekly food intake in male mice, with data represented as means ± standard deviation with individual data points. (B) Grip strength measurements (gram force) in male and female mice, with dots representing the average values and error envelopes as standard deviation. No significant differences in grip strength between males and females were observed. Food intake (*p* = 0.0801) and grip strength (*p* = 0.0005) were compared using one‐way repeated‐measures ANOVAs with Tukey's post hoc testing (*p < 0.05 compared to day −1).

**Figure S3:** Automated muscle cross‐sectional area analysis using MIRA Vision. Representative images of (A) input immunofluorescent image of a laminin‐stained muscle cross‐section and (B) MIRA Vision's segmentation of individual fibres for analysis.

**Figure S4:** Live/dead FACS sort prior to single‐cell RNA sequencing. Representative plots for gating strategy to sort for live cells (Calcein AM^+^SYTOX‐AAD^−^) prior to scRNAseq preparation. Flow plots output by the Ottawa Hospital Research Institute Flow Cytometry Core following FACS.

**Figure S5:** Muscle panel gating strategy and fluorescence‐minus‐one controls. Fluorescence‐minus‐one (FMO) flow plots for (A) muscle cell flow cytometry panel [All cells ➔ Singlets ➔ Live cells (SYTOX AAD^−^) ➔ CD31 vs. CD45 ➔ (CD31^−^CD45^−^) PDGFRα vs. ITGA7] and (B) immune cell panel for myeloid populations [All cells ➔ Singlets ➔ Live cells (LIVE DEAD Blue^−^) ➔ CD45^+^ ➔ CD11b^+^, CD11c^+^ ➔ (from CD11b^+^) Ly6C vs. Ly6G ➔ (from Ly6C^low/high^) F4/80 vs. CD206] and lymphocyte populations [CD45^+^ ➔ CD19^+^ ➔ (from CD19^−^) NK1.1^+^ ➔ (from NK1.1^−^) CD3e^+^]. Plot titles indicate the cells that were previously gated on, and x and y axes indicate the channels used for gating.

**Figure S6:** Satellite cell isolation purity with MACS. Cells were depleted with CD31, CD45 and PDGFRα microbeads, then positively selected for ITGA7 with MACS. Cells were then either cytospun for 10 min at 600 g directly onto slides for fixation and staining or cultured on Matrigel‐coated slides for 30 min prior to fixation and staining with PAX7 and DAPI. (A) Freshly isolated, cytospun cells, imaged at 20× magnification were 69.7% (±8.6%) PAX7+ (*n* = 3). (B) Cells cultured on Matrigel for 30 min, imaged at 40× magnification were 95.2% (±6.6%) PAX7+ (*n* = 10).

**Figure S7:** Quality control measures for scRNAseq data. Violin plots for (A) number of counts, (B) number of features and (C) percentage of mitochondrial genes per cell and separated by time point. (D) Scatterplot comparing the number of features (x axis) to the number of counts (y axis) with a line of best fit and Pearson's coefficient at each time point. (E) Violin plots comparing the number of counts and (F) number of features in cells identified as singlets and doublets by scDblFinder, separated by time point. (G) UMAP plots highlighting the identified doublets.

**Figure S8:** scRNAseq data integration and CaSC subcluster validation. (A) UMAP plots highlighting MuSCs from C26 and LLC models to visually demonstrate their integration. (B‐C) Violin plots of cachexia scores for (B) MuSCs subclusters and (C) conditions of integrated MuSCs. (D) Violin plots for cachexia scores for MuSCs in models of (left to right) aging,^20^ denervation,^21^ irradiation,^9^ muscular dystrophy^22^ and regeneration.^10^ (E) Upregulated genes in MuSCs from cachectic PDAC patients compared to noncancer and weight‐stable controls.

**Figure S9:** Flow cytometry analyses of immune cell changes in the spleen. (A) Spleen mass at each time point. (B) Representative flow cytometry plots and gating strategies for immune cells in the spleen, including myeloid populations [Myeloid cells ➔ Singlets ➔ Live cells (LIVE DEAD Blue^−^) ➔ CD45^+^ ➔ CD11b^+^, CD11c^+^ ➔ (from CD11b^+^) Ly6C vs. Ly6G ➔ (from Ly6C^low/high^) F4/80 vs. CD206] and lymphocyte populations [Lymphocyte cells ➔ Singlets ➔ Live cells (LIVE DEAD Blue^−^) ➔ CD45^+^ ➔ CD19^+^ ➔ (from CD19^−^) NK1.1^+^ ➔ (from NK1.1^−^) CD3e^+^], with plot titles indicating the cells that were previously gated on. (C) Immune cell counts per spleen mass. Data are represented by means ± standard deviation with individual data points (● males, ◆ females) and were compared using a one‐way ANOVA (*p < 0.05, **p < 0.01, ***p < 0.001, ****p < 0.0001).

**Figure S10:** Flow cytometry analyses of immune cell changes in the tumour. (A) Tumour mass at each time point. (B) Representative flow cytometry plots and gating strategies for immune cells in the tumour, including, myeloid populations [All cells ➔ Singlets ➔ Live cells (LIVE DEAD Blue^−^) ➔ CD45^+^ ➔ CD11b^+^, CD11c^+^ ➔ (from CD11b^+^) Ly6C vs. Ly6G ➔ (from Ly6C^low/high^) F4/80 vs. CD206] and lymphocyte populations [CD45^+^ ➔ CD19^+^ ➔ (from CD19^−^) NK1.1^+^ ➔ (from NK1.1^−^) CD3e^+^] with plot titles indicating the cells that were previously gated on. (C) Immune cell counts per tumour mass. Data are represented by means ± standard deviation with individual data points (● males, ◆ females) and were compared using a one‐way ANOVA (**p < 0.01, ****p < 0.0001).

**Figure S11:** Endothelial cell and FAPs annotation analyses. (A) UMAP plot of all cells highlighting endothelial cells. (B) Dotplot of unique markers for endothelial cell subclusters. (C) GO terms of downregulated genes in all cells at 2, 2.5 and 3.5 weeks compared to sham. (D) UMAP plot of all cells highlighting FAPs. (E) Violin plots for unique genes expressed in FAPs subclusters. (F) UMAP plot of pseudotime analysis in FAPs subclusters.

**Figure S12:** Markers of cellular senescence in CaSCs. (A) Enriched KEGG pathways for upregulated genes in the integrated CaSC subcluster related to cellular senescence. (B) Violin plots for significantly upregulated genes enriched in the integrated CaSC subcluster and identified in the cellular senescence KEGG pathway. (C) Violin plot for Senescence Score between integrated MuSC subclusters, which was performed from genes in msigdbr database, CP:REACTOME, REACTOME_CELLULAR_SENESCENCE and scored using UCell. (D) SA‐β‐galactosidase staining in C2C12 myoblasts treated with conditioned media from C2C12 myoblasts or LLC cells for 48 h.

**Figure S13:** Increased MuSC proliferation in cancer cachexia following muscle injury. (A) Representative, immunofluorescent image for PAX7, EdU, laminin and DAPI. PAX7^+^EdU^+^ cells per area in uninjured and 2‐day postcardiotoxin injury in sham and 3.5‐week tumour‐bearing, male mice imaged at 20× objective, with a scale bar of 100 μm and arrows pointing to a PAX7^+^EdU^+^ cell. (B) PAX7^+^EdU^+^ cells per area in uninjured muscle and (C) at 2‐day postcardiotoxin injury. Sham and 3.5‐week tumour‐bearing mice were compared using an unpaired *t*‐test.

**Data S1:** Supporting Information.

**Data S2:** Supporting Information.

**Data S3:** Supporting Information.

**Data S4:** Supporting Information.

**Data S5:** Supporting Information.

**Data S6:** Supporting Information.

**Data S7:** Supporting Information.

**Data S8:** Supporting Information.

**Table S1:** Key resources.
**Table S2:** Immunofluorescence staining antibodies.
**Table S3:** Flow cytometry antibodies and reagents.
**Table S4:** Magnetic beads‐conjugated antibodies for MACS.
**Table S5:** Software and algorithms.
**Table S6:** R packages and versions.
**Table S7:** Complete blood cell counts.

## Data Availability

Codes and data are available on GitHub (https://github.com/musclesci/CachexiaMuscleTimecourse.git).
