# Supplementary figures and images for: Single‐Cell RNA‐Sequencing Reveals Cachectic Satellite Cell Population in Muscle of Male Mice With Cancer Cachexia
Source: J Cachexia Sarcopenia Muscle. 2026 Mar 27;17(2):e70260. doi: 10.1002/jcsm.70260 (PMC13140835; doi:10.1002/jcsm.70260)

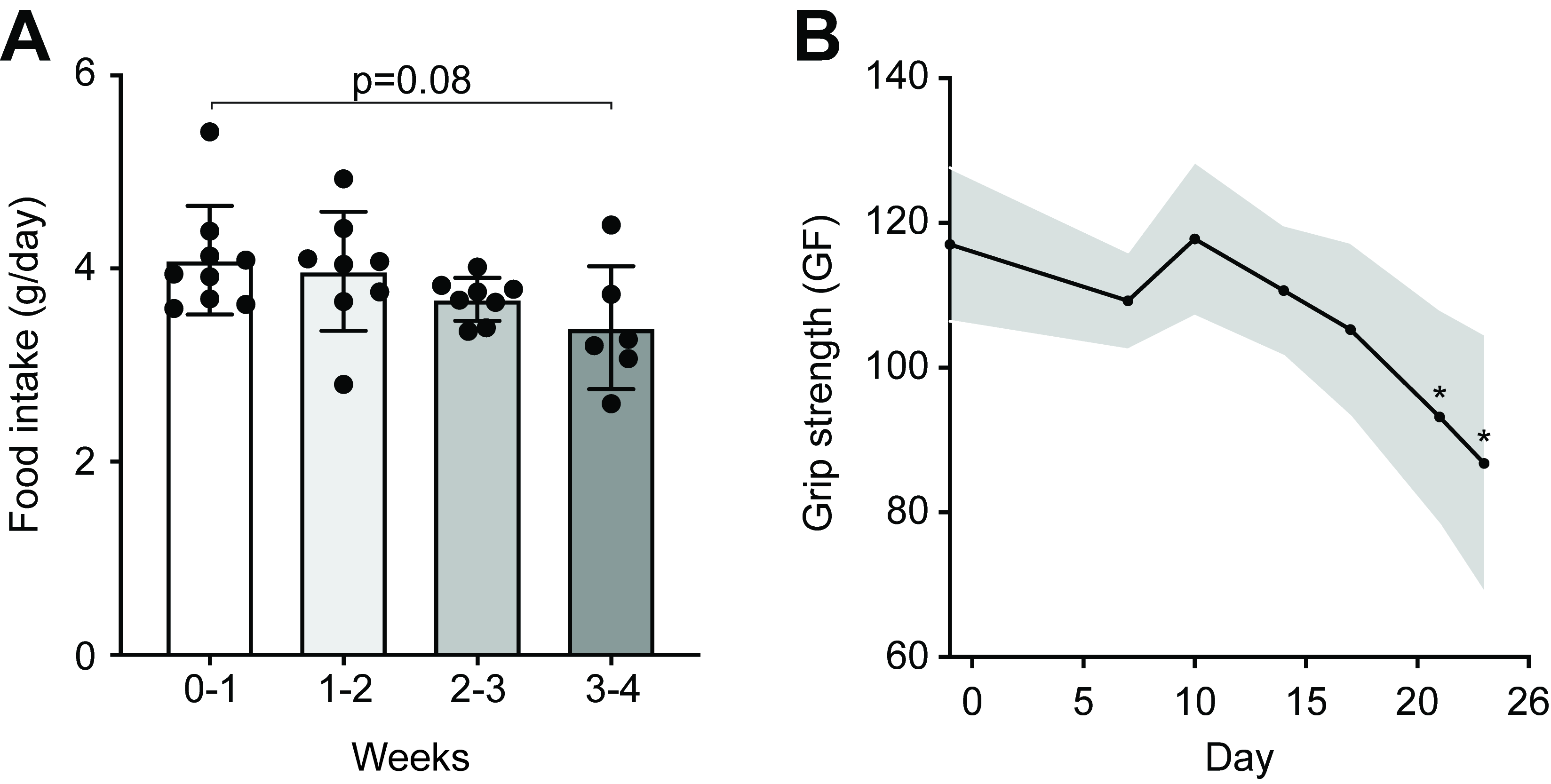

Supplement: Supplementary file 2 — Figure S2: Food consumption and grip strength over the course of cancer cachexia in male mice. (A) Weekly food intake in male mice, with data represented as means ± standard deviation with individual data points. (B) Grip strength measurements (gram force) in male and female mice, with dots representing the average values and error envelopes as standard deviation. No significant differences in grip strength between males and females were observed. Food intake (p = 0.0801) and grip strength (p = 0.0005) were compared using one‐way repeated‐measures ANOVAs with Tukey's post hoc testing (*p < 0.05 compared to day −1). [file JCSM-17-e70260-s019.tif]

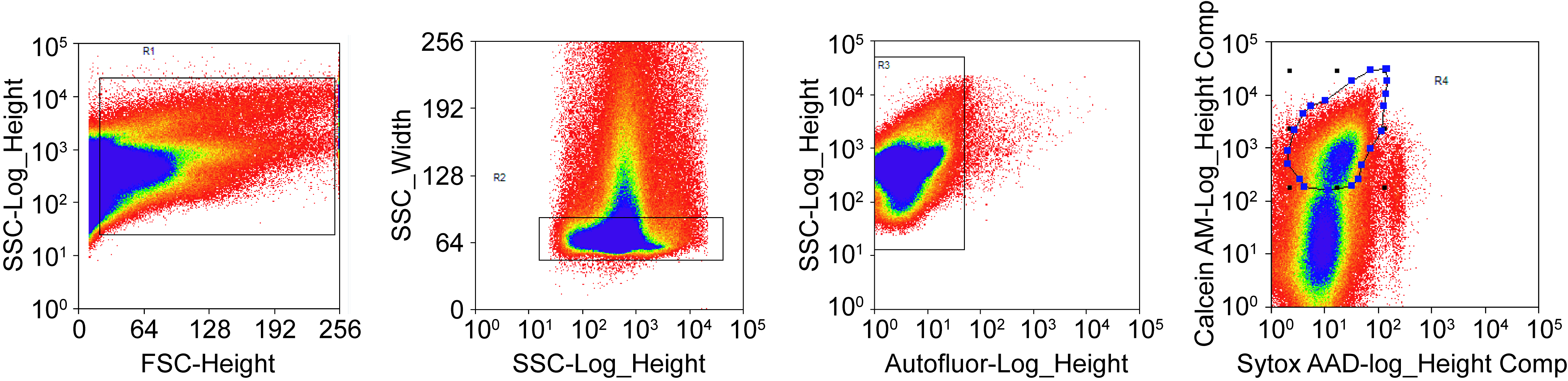

Supplement: Supplementary file 4 — Figure S4: Live/dead FACS sort prior to single‐cell RNA sequencing. Representative plots for gating strategy to sort for live cells (Calcein AM+SYTOX‐AAD−) prior to scRNAseq preparation. Flow plots output by the Ottawa Hospital Research Institute Flow Cytometry Core following FACS. [file JCSM-17-e70260-s005.tif]

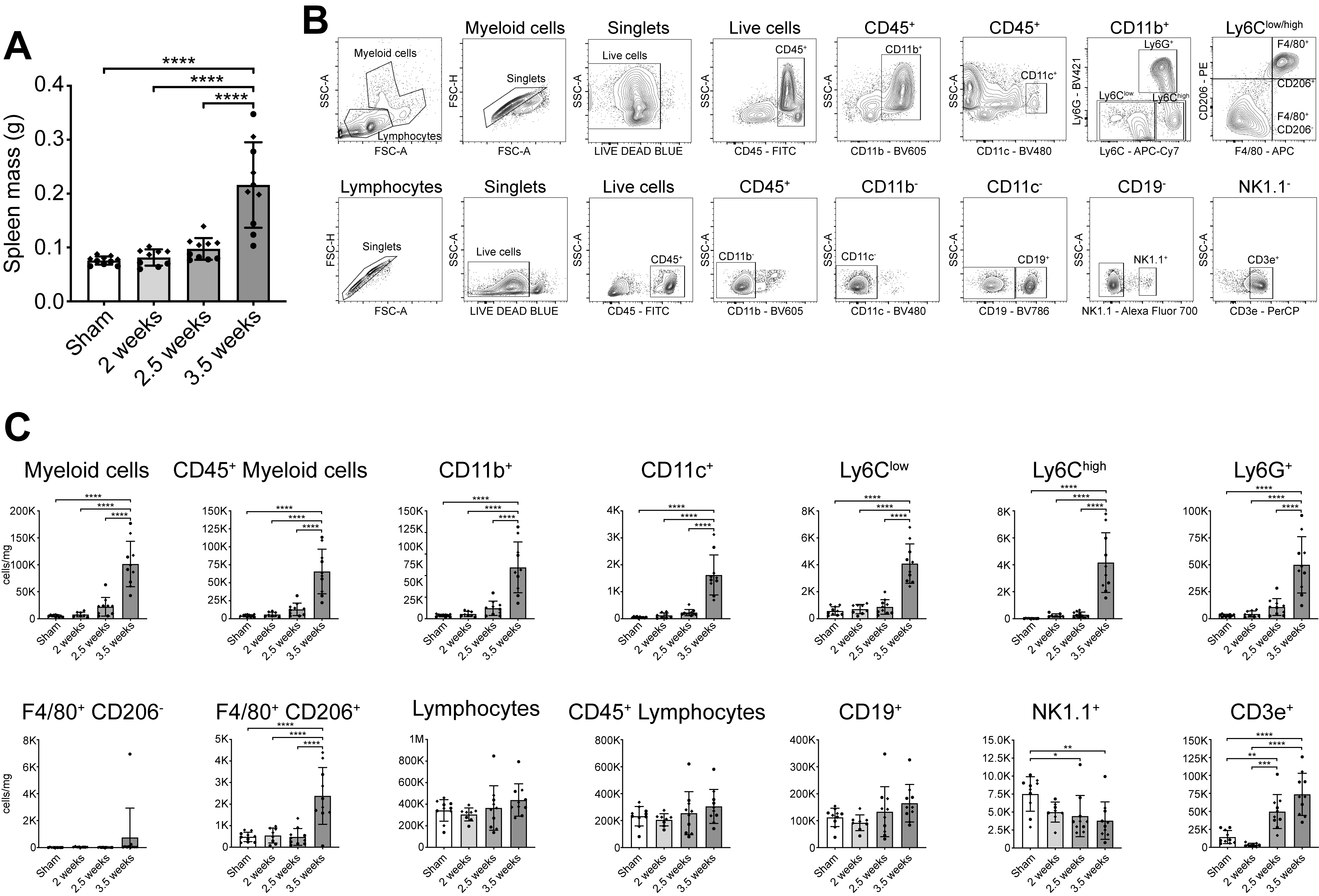

Supplement: Supplementary file 9 — Figure S9: Flow cytometry analyses of immune cell changes in the spleen. (A) Spleen mass at each time point. (B) Representative flow cytometry plots and gating strategies for immune cells in the spleen, including myeloid populations [Myeloid cells ➔ Singlets ➔ Live cells (LIVE DEAD Blue−) ➔ CD45+ ➔ CD11b+, CD11c+ ➔ (from CD11b+) Ly6C vs. Ly6G ➔ (from Ly6Clow/high) F4/80 vs. CD206] and lymphocyte populations [Lymphocyte cells ➔ Singlets ➔ Live cells (LIVE DEAD Blue−) ➔ CD45+ ➔ CD19+ ➔ (from CD19−) NK1.1+ ➔ (from NK1.1−) CD3e+], with plot titles indicating the cells that were previously gated on. (C) Immune cell counts per spleen mass. Data are represented by means ± standard deviation with individual data points (● males, ◆ females) and were compared using a one‐way ANOVA (*p < 0.05, **p < 0.01, ***p < 0.001, ****p < 0.0001). [file JCSM-17-e70260-s004.tif]

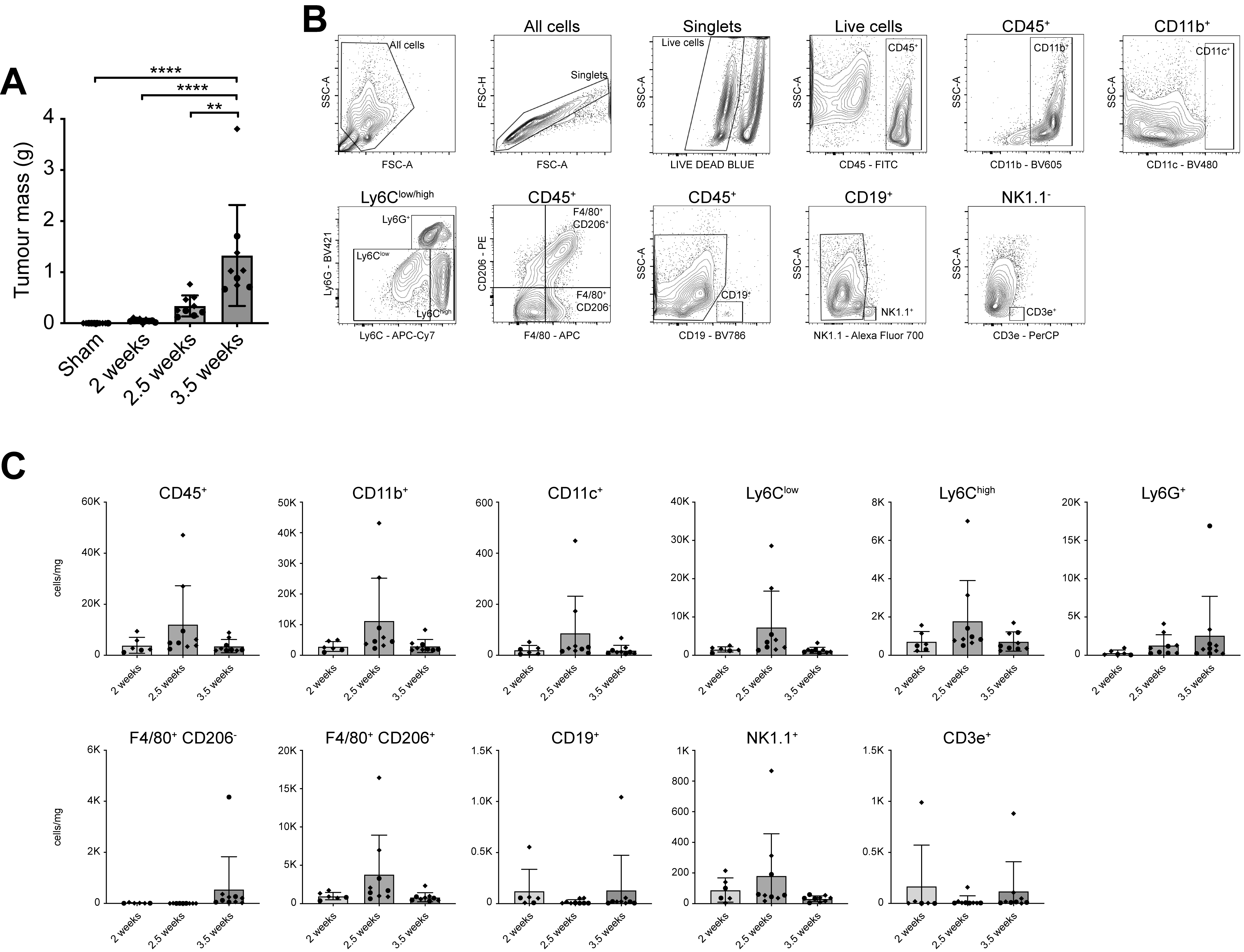

Supplement: Supplementary file 10 — Figure S10: Flow cytometry analyses of immune cell changes in the tumour. (A) Tumour mass at each time point. (B) Representative flow cytometry plots and gating strategies for immune cells in the tumour, including, myeloid populations [All cells ➔ Singlets ➔ Live cells (LIVE DEAD Blue−) ➔ CD45+ ➔ CD11b+, CD11c+ ➔ (from CD11b+) Ly6C vs. Ly6G ➔ (from Ly6Clow/high) F4/80 vs. CD206] and lymphocyte populations [CD45+ ➔ CD19+ ➔ (from CD19−) NK1.1+ ➔ (from NK1.1−) CD3e+] with plot titles indicating the cells that were previously gated on. (C) Immune cell counts per tumour mass. Data are represented by means ± standard deviation with individual data points (● males, ◆ females) and were compared using a one‐way ANOVA (**p < 0.01, ****p < 0.0001). [file JCSM-17-e70260-s001.tif]

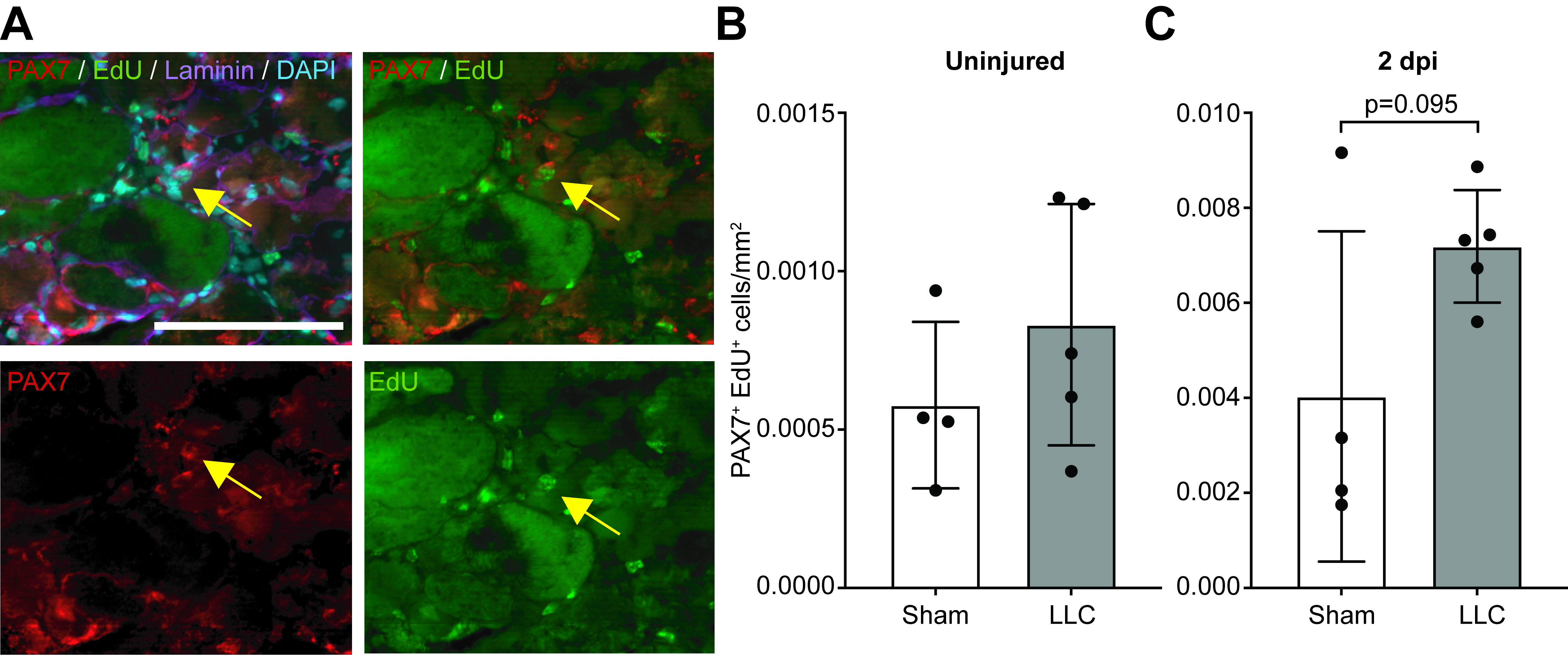

Supplement: Supplementary file 13 — Figure S13: Increased MuSC proliferation in cancer cachexia following muscle injury. (A) Representative, immunofluorescent image for PAX7, EdU, laminin and DAPI. PAX7+EdU+ cells per area in uninjured and 2‐day postcardiotoxin injury in sham and 3.5‐week tumour‐bearing, male mice imaged at 20× objective, with a scale bar of 100 μm and arrows pointing to a PAX7+EdU+ cell. (B) PAX7+EdU+ cells per area in uninjured muscle and (C) at 2‐day postcardiotoxin injury. Sham and 3.5‐week tumour‐bearing mice were compared using an unpaired t‐test. [file JCSM-17-e70260-s012.tif]
