# Supplementary material for: Single‐Cell RNA‐Sequencing Reveals Cachectic Satellite Cell Population in Muscle of Male Mice With Cancer Cachexia
Source: J Cachexia Sarcopenia Muscle. 2026 Mar 27;17(2):e70260. doi: 10.1002/jcsm.70260 (PMC13140835; doi:10.1002/jcsm.70260)
Supplement: Supplementary file 20 — Data S7: Supporting Information. [file JCSM-17-e70260-s018.docx]

**SUPPLEMENTARY METHODS**

*Food intake*

As mice were housed individually, food intake was measured by weighing food at the beginning and the end of the week (or in the case of after 3 weeks, the start of the week until collections), taking the difference, and dividing by the number of days between measurements (Supplementary Figure 2A).

*Grip Strength*

Forelimb grip strength measurements were performed using the Chatillon DFE II Grip Meter (Columbus Instruments) at the University of Ottawa Behavioural Core (Supplementary Figure 2B). Male and female mice were tested on multiple days prior to inoculation (day -1) and following inoculation (days 3, 7, 10, 14, 17, 21, 23). Mice were acclimatized in the room for 30 minutes prior to experimentation. For each mouse per experimental day, 6 grip measurements were taken, where the top measurement and bottom measurements were removed and the remaining 4 were averaged to obtain the final value.

*Immunofluorescence*

Immunofluorescent staining protocols are in the “Stain protocols” supplementary file. Antibody information is in Supplementary Table S2.

Imaging was performed on the Axio Observer7 microscope with the Axiocam 705 color camera (Zeiss) using the Zen imaging software, and images were quantified using FIJI. Muscle cross-sectional area analyses were automated using the MIRA Vision platform (Supplementary Figure 3). Investigators were blinded for all manual analyses.

*Muscle collection and single-cell preparation*

Left *gastrocnemius/soleus* complex muscles were embedded in O.C.T., frozen in liquid nitrogen-cooled isopentane, and stored at -80°C. Muscles were sectioned using a cryostat at 7 μm and stored at -80°C prior to staining.

To prepare single-cell solutions of skeletal muscle cells, muscles were dissected free of tendons and adipose tissue and placed in gentleMACS C tubes containing 5 mL (whole hindlimb muscles) or 2 mL (individual muscles, right gastrocnemius/soleus complex for muscle populations, right quadricep for immune populations) of enzymatic solution (Ham’s F-10 media with 0.01 g/mL Collagenase B, 0.004 g/mL Dispase II, and 0.5 Kunitz units/mL of DNAse, 0.22 μm-filtered and warmed to 37°C). Muscles were manually digested with scissors until a homogenous slurry was observed, then homogenized using the gentleMACS Octo Dissociator. Dissociated muscles were re-suspended in an equal volume of FBS and filtered through FBS-coated 100 μm, 70 μm, and 40 μm filters. Each filter was washed with PBS, and the solution was centrifuged at 600 g for 10 minutes. After the supernatant was removed, the pellet was resuspended with 400 μL (whole hindlimbs) or 100 μL (individual muscles) of Red Blood Cell Lysis Buffer and incubated at room temperature for 5 minutes. The solution was then resuspended in 10 mL of FACS buffer (1X PBS with 10% FBS, 2 mM EDTA, 0.22 μm-filtered and cooled to 4°C) or MACS buffer (1X PBS with 5 mg/mL bovine serum albumin, 2 mM EDTA, 0.22 μm-filtered and cooled to 4°C) and centrifuged again at 600 g for 10 minutes, after which the pellet was used for downstream applications.

*Flow cytometry*

FACS and flow cytometry antibodies and reagents are in Supplementary Table S3.

A muscle-specific panel (SYTOX AAD, CD31 – BV421, CD45 – BV785, ITGA7 – APC, PDGFRα - BV605) and immune cell panel (LIVE/DEAD Blue, CD45 – FITC, CD11b – BV605, CD11c – BV480, Ly6C – APC-Cy7, Ly6G – BV421, F4/80 – APC, CD206 – PE, CD3e – PerCP, CD19 – AF700, NK1.1 – BV786) for muscle, spleen, and tumours were used for flow cytometry. Following isolation of single-cell suspensions, cells were resuspended in 200 μL FACS buffer (muscle panel) or 1X PBS (immune panel) and stained with respective antibodies for 30 minutes at 4°C in the dark. After 30 minutes, cells were washed with 1 mL of buffer, centrifuged, re-suspended in 200 μL of buffer and stained for viability markers for 15 minutes at 4°C. Cells were then washed again, centrifuged, resuspended in 200 μL of buffer and processed for flow cytometry.

For the muscle panel, the PDGFRα antibody was added first, stained for 30 minutes, washed, centrifuged, re-suspended in FACS buffer, then proceeded with other antibody staining. For the immune panel, True-stain monocyte and Fc-receptor blocks were incubated for 10 minutes prior to staining, then proceeded directly to cell staining.

Flow cytometry was run on the Cytek Aurora Cytometer using the SpectroFlo software. FCS files were processed for cell counts and graphs were made with FlowJo. Colours were unmixed using single-colour stains on compensation beads and gates were established using single colour and fluorescence minus one controls (Supplementary Figure 5).

*Magnetic-activated cell sorting (MACS) and culture of muscle satellite cells*

Following muscle hindlimb isolation and single-cell suspension, pellets were resuspended in 70 μL of MACS buffer and incubated with 10 μL each of CD31, CD45, and PDGFRα-conjugated microbeads (Supplementary Table S4) for 15 minutes at 4°C in the dark. Cell suspensions were washed with 1 mL of MACS buffer and centrifuged at 1 200 g for 10 minutes. The pellet was re-suspended in 1 mL of MACS buffer and run through an LD column. The flow-through was centrifuged at 600 g for 10 minutes, after which the cell pellet was re-suspended in 60 μL of MACS buffer and incubated with 40 μL of ITGA7-conjugated microbeads for 15 minutes, washed with 1 mL of MACS buffer and centrifuged at 1 200 g for 10 minutes. The cell pellet was re-suspended in 1 mL of MACS buffer and run through an LS column. Once the liquid had run through, columns were taken off the magnetic stand, filled with 5 mL of MACS buffer, plunged and repeated to elute the ITGA7+ cells. To culture ITGA7+ cells, eluted cells were centrifuged and the cell pellet was resuspended in primary satellite cell culture medium (DMEM with 20% FBS, 5 ng/mL of bFGF, 1% penicillin/streptomycin, 0.22 μm-filtered and warmed to 37°C) and plated in Matrigel-coated 8-well chamber slides. Myogenic cell purity was tested via immunofluorescent staining with PAX7 and found to have 69.7±8.6% PAX7+ cells immediately following isolation, and 95.2±6.5% after culturing for 30 minutes (Supplementary Figure 6).

*MuSC cell size and circularity*

MuSCs were isolated from Sham and 3.5-week tumour-bearing mice via MACS and cultured for 30 minutes on Matrigel-coated 8-well chamber slides. MuSCs were then fixed, stained and imaged at 40X objective. PAX7^+^ cells were manually circled on FIJI for cell size and circularity measurements.

*Bioinformatic analyses*

R packages and versions are in Supplementary Table S6. scRNAseq data were processed in R Studio using a standard Seurat pipeline.^1^ 10,934 Sham, 12,436 2-weeks, 10,538 2.5-weeks, and 10,429 3.5-weeks were sequenced. Low-quality cells (>10% mitochondrial genes, 200>nFeatures>60,000, 500>nCounts>15,000) were removed (Supplementary Figure 7). Data were then normalized (scale factor of 10,000), the top variable features (2,000) found, scaled, and principal component analysis was run. Cell identities were determined (FindNeighbors, FindClusters, RunUmap) and scDblFinder was used to identify and remove doublets, then each condition was combined into a single Seurat object using SelectIntegrationFeatures, FindIntegrationAnchors (to correct for sample differences), and IntegrateData functions. The combined object was re-processed, and cell types were annotated by the top expressed genes using the FindAllMarkers function. Cells annotated as “Erythrocytes” and “Epithelial cells” were removed from analyses due to not being natively found in skeletal muscle. After filtering, 7,793 Sham, 8,565 2-weeks, 6,824 2.5-weeks, and 6,124 3.5-weeks cells were used for downstream analyses. MuSC data was integrated with the C26 dataset^2^ using Harmony to correct for batch effects, then proceeded with downstream analyses.

Codes for pre-processing and downstream bioinformatic analysis can be found on GitHub (<https://github.com/musclesci/CachexiaMuscleTimecourse.git>), including pseudo-bulk analysis, differential gene expression using FindMarkers and FindAllMarkers functions, cell subclustering, Monocle3 pseudotime analyses,^3^ GO terms and KEGG pathway analyses, cell communication via CellChat,^4^ and cell scoring via UCell.^5^ Gene lists for Cachexia scores (analyses in Supplementary Figure 8) and for GO terms and KEGG pathway analyses can be found in the MuSCs_DEGs supplementary file. The gene list for Cell Matrix Adhesion scoring can be found using MSigDB-R (GOBP_CELL_MATRIX_ADHESION).^6,7^

*Single myofibre isolation and culture*

Single *Extensor digitorium longus* (EDL) myofibes were isolated from Sham and 3.5-week tumour-bearing mice as previously described (Brun et al., 2018).^8^ Briefly, EDLs were isolated with tendons preserved and incubated in EDL-dissociation solution (DMEM with 2 mg/mL of collagenase I, 0.22 μm-filtered and warmed to 37°C) for 45 minutes followed by manual myofibre dissociation with a wide-mouth Pasteur pipette, three subsequent washes (in DMEM with 1% penicillin/streptomycin, 0.22 μm-filtered and warmed to 37°C) and either fixed immediately in warm, 4% PFA (37°C for 15 minutes) or cultured for 24 hours (in DMEM with 20% FBS, 1% chicken embryo extract, 2.5 ng/mL of bFGF, 0.22 μm-filtered and warmed to 37°C). Myofibres were fixed and stained in wells of 6-well culture plates, dried on a slide, and mounted with coverslips for imaging at 20X magnification.

*Spleen collection and single-cell preparations*

Spleens were isolated and placed in a 100 μm filter in a 10 cm cell culture dish with 10 mL of cold PBS. Spleens were manually digested by gradually grinding the tissue with the back of a syringe plunger through the filter. The solution was centrifuged at 600 g for 10 minutes, after which the pellet was incubated with 500 μL of Red Blood Cell Lysis buffer for 5 minutes at room temperature, resuspended in 10 mL of FACS buffer, centrifuged at 600 g for 10 minutes again, and resuspended in 1 mL of PBS. The sample was then further diluted by adding 20 μL of the spleen solution to 180 μL of PBS (which was previously determined to be approximately 1 000 000 cells) and stained for the immune cell flow cytometry panel and respective controls. Analyses can be found in Supplementary Figure 9.

*Tumour collection and single-cell preparations*

Tumours were digested using a tumour dissociation kit according to manufacturer’s instructions. One gram of tumour was weighed and cut into 2-4 mm pieces, then added to gentleMACS C tubes containing 2.35 mL of DMEM. Enzymes D (100 μL), R (25 μL), and A (12.5 μL) were added to the solution and digested on the gentleMACS Octo Dissociator. Tumour digests were filtered through 100 μm and 40 μm filters and washed with 10 mL of DMEM. The solution was centrifuged at 300 g for 7 minutes, after which the cell pellet was re-suspended in 1 mL of Red Blood Cell Lysis Buffer for 1 minute. An additional 14 mL of cold PBS was added to the pellet and centrifuged again at 300 g for 7 minutes. The pellet was then re-suspended in 15 mL of cold PBS, centrifuged again at 300 g for 7 min, re-suspended in cold PBS (1 mL for 2-week and 2.5-week tumours, 2 mL for 3.5-week tumours), and stained for the immune cell flow cytometry panel and respective controls. Analyses can be found in Supplementary Figure 10.

*Complete blood cell counts*

Immediately following euthanasia, cardiac puncture was performed to extract blood, was moved into EDTA-coated Microvette tubes and gently inverted to prevent blood clotting. Samples were then shipped on ice to IDEXX BioAnalytics (North Grafton, Massachusetts) to perform whole blood cell counts. Analyses can be found in Supplementary Table S7.

*C2C12 myoblast conditioned media and SA-β-galactosidase analysis*

C2C12s (2,500 cells/cm^2^) and LLC cells (14,000 cells/cm^2^) were seeded and cultured for 48 hours in DMEM with 10% FBS and 1% penicillin/streptomycin, after which media was changed, and cells were allowed to grow for an additional 48 hours. C2C12 myoblasts were seeded at 2,500 cells/cm^2^ and allowed to adhere for 12 hours prior to collection of conditioned media. Conditioned media was then collected from C2C12 (control) and LLC cells, spun at 300 g for 5 min, and filtered using a 0.22 μm filter. Conditioned media was added to adhered C2C12 myoblasts in a 1:1 ratio with fresh growth media for 48 hours, after which cells were collected and stained for SA-β-galactosidase according to manufacturer’s instructions. Cells were imaged at 10X magnification in 5 random locations throughout the well, and were analyzed by the percentage of SA-β-galactosidase+ cells per condition (Supplementary Figure 12).

*Muscle injuries*

Subcutaneous buprenorphine (0.1 mg/kg) was administered 1 hour prior to injury. Mice were injected with 50 μL of 10 μM cardiotoxin into the left *tibialis anterior*, where the right remained uninjured. Cardiotoxin injuries were performed at 3.5-weeks post-inoculation and muscle was collected after 2 days.

*EdU administration*

EdU was administered via intraperitoneal injection (4 μg/g of body weight), 18 hours prior to muscle collection. EdU positive cells were visualized in muscle cross sections according to manufacturer’s instructions (Supplementary Figure 13).

*Supplementary References*

1. Stuart T, Butler A, Hoffman P, Hafemeister C, Papalexi E, Mauck WM *et al.* Comprehensive Integration of Single-Cell Data. *Cell* 2019;**177**:1888-1902.e21.

2. Pryce BR, Oles A, Talbert EE, Romeo MJ, Vaena S, Sharma S *et al.* Muscle inflammation is regulated by NF-κB from multiple cells to control distinct states of wasting in cancer cachexia. *Cell Reports* 2024;**43**:114925.

3. Trapnell C, Cacchiarelli D, Grimsby J, Pokharel P, Li S, Morse M *et al.* The dynamics and regulators of cell fate decisions are revealed by pseudotemporal ordering of single cells. *Nat Biotechnol* 2014;**32**:381–386.

4. Jin S, Guerrero-Juarez CF, Zhang L, Chang I, Ramos R, Kuan C-H *et al.* Inference and analysis of cell-cell communication using CellChat. *Nat Commun* 2021;**12**:1088.

5. Andreatta M, Carmona SJ. UCell: Robust and scalable single-cell gene signature scoring. *Computational and Structural Biotechnology Journal* 2021;**19**:3796–3798.

6. Castanza AS, Recla JM, Eby D, Thorvaldsdóttir H, Bult CJ, Mesirov JP. Extending support for mouse data in the Molecular Signatures Database (MSigDB). *Nat Methods* 2023;**20**:1619–1620.

7. Subramanian A, Tamayo P, Mootha VK, Mukherjee S, Ebert BL, Gillette MA *et al.* Gene set enrichment analysis: A knowledge-based approach for interpreting genome-wide expression profiles. *Proc Natl Acad Sci USA* 2005;**102**:15545–15550.

8. Brun CE, Wang YX, Rudnicki MA. Single EDL Myofiber Isolation for Analyses of Quiescent and Activated Muscle Stem Cells. In: Lacorazza HD, editor. *Cellular Quiescence*. Springer New York: New York, NY; 2018. pp. 149–159.
