# Supplementary material for: Single‐Cell RNA‐Sequencing Reveals Cachectic Satellite Cell Population in Muscle of Male Mice With Cancer Cachexia
Source: J Cachexia Sarcopenia Muscle. 2026 Mar 27;17(2):e70260. doi: 10.1002/jcsm.70260 (PMC13140835; doi:10.1002/jcsm.70260)
Supplement: Supplementary file 21 — Data S8: Supporting Information. [file JCSM-17-e70260-s020.docx]

**LAMININ, MUSCLE CROSS SECTIONS.**

| **Marker** | **Species** | **Dilution** | **Antibody information** | **Secondary conjugation** | **Secondary antibody information** |
| --- | --- | --- | --- | --- | --- |
| Laminin | Rabbit | 1:250 | Abcam ab11575 | Alexa-fluor 488 donkey anti-rabbit | Jackson Immuno-Reseach, 711-545-152 |
| DAPI |  | 0.5 μg/mL | ThermoFisher Scientific, D3571 |  |  |

| **Step** | **Instructions** | **Time / Temperature** | **Materials** | **Notes** |
| --- | --- | --- | --- | --- |
| 1 | Dry slides | 5 min / RT |  |  |
| 2 | Fix | 15 min / RT | 4% PFA |  |
| 3 | Wash | 3x5 min / RT | 1X PBS |  |
| 4 | Permeabilize | 15 min / RT | 0.5% Triton X-100 |  |
| 5 | Wash | 3x5 min / RT | 1X PBS |  |
| 6 | Primary antibody | ON / 4°C | Laminin in 0.1% Triton X-100 |  |
| 7 | Wash | 3x5 min / RT | 0.1% Triton X-100 |  |
| 8 | Secondary antibody | 1 h / RT | 488 anti-rabbit 1:500 in 0.1% Triton X-100 | Everything conducted in the dark going forward |
| 9 | Wash | 3x5 min / RT | 0.1% Triton X-100 |  |
| 10 | DAPI | 5 min / RT | DAPI 1:20 000 in 1X PBS |  |
| 11 | Wash | 3x5 min / RT | 1X PBS |  |
| 12 | Dry | 15-30 min / RT |  |  |
| 13 | Mount and coverslip |  | Fluorescence mounting media and coverslip |  |

**PAX7/Ki67/LAMININ, MUSCLE CROSS-SECTIONS.**

| **Marker** | **Species** | **Dilution** | **Antibody information** | **Secondary conjugation** | **Secondary antibody information** | **Tertiary antibody** | **Tertiary antibody information** |
| --- | --- | --- | --- | --- | --- | --- | --- |
| PAX7 | Mouse | 1:100 | DSHB, PAX7-c | Biotin donkey anti-mouse | Invitrogen, 715-066-150 | Cy3-conjugated streptavidin | Jackson Immuno-Research, 016-160-084 |
| Ki67 | Rat | 1:200 | Invitrogen, 14-5698-82 | Alexa-fluor 488 donkey anti-rat | Invitrogen, A21208 |  |  |
| Laminin | Rabbit | 1:250 | Abcam, ab11575 | Alexa-fluor 647 goat anti-rabbit | Jackson Immuno-Research, 111-605-003 |  |  |
| DAPI |  | 0.5 μg/mL | ThermoFisher Scientific, D3571 |  |  |  |  |

| **Step** | **Instructions** | **Time / Temperature** | **Materials** | **Notes** |
| --- | --- | --- | --- | --- |
| 1 | Dehydrate slides | 30 min / 60°C | Heat block |  |
| 2 | Fix | 15 min / RT | 4% PFA |  |
| 3 | Wash | 3x5 min / RT | 1X PBS |  |
| 4 | Antigen retrieval | 30 min / 90°C water bath | Citrate buffer | 900 mL ddH_2_O, 1.92 g citric acid, pH to 6.0 with NaOH, 5 mL Tween 20, top to 1000 mL |
| 5 | Antigen retrieval 2 | 30 min / RT | Citrate buffer | Take out of water bath and leave for 30 min |
| 6 | Wash | quick | 1X PBS |  |
| 7 | Permeabilize | 15 min / RT | 0.5% Triton X-100 |  |
| 8 | Wash | 3x5 min / RT | 1X PBS |  |
| 9 | Block | 1 h / RT | 0.1% Triton X-100, 2.5% DS, 2.5% GS, MoM block |  |
| 10 | Wash | 3x5 min / RT | 0.1% Triton X-100 |  |
| 11 | Primary antibodies | ON / 4°C | PAX7, Ki67, Laminin in block |  |
| 12 | Wash | 3x5 min / RT | 0.1% Triton X-100 |  |
| 13 | Secondary antibodies | 1 h / RT | Biotin anti-mouse 1:250, 488 anti-rat 1:2 000, 647 anti-rabbit 1:500 in 0.1% Triton X-100 | Everything conducted in the dark going forward |
| 14 | Wash | 3x5 min / RT | 0.1% Triton X-100 |  |
| 15 | Tertiary antibody | 1 h / RT | Cy3-conjugated streptavidin 1:250 in 0.1% Triton X-100 |  |
| 16 | Wash | 3x5 min / RT | 0.1% Triton X-100 |  |
| 17 | DAPI | 5 min / RT | DAPI 1:20 000 in 1X PBS |  |
| 18 | Wash | 3x5 min / RT | 1X PBS |  |
| 19 | Dry | 15-30 min / RT |  |  |
| 20 | Mount and coverslip |  | Fluorescence mounting media and coverslip |  |

**PAX7/EdU, MUSCLE CROSS SECTIONS.**

| **Marker** | **Species** | **Dilution** | **Antibody information** | **Secondary conjugation** | **Secondary antibody information** | **Tertiary antibody** | **Tertiary antibody information** |
| --- | --- | --- | --- | --- | --- | --- | --- |
| PAX7 | Mouse | 1:100 | DSHB, PAX7-c | Biotin donkey anti-mouse | Invitrogen, 715-066-150 | Cy3-conjugated streptavidin | Jackson Immuno-Research, 016-160-084 |
| EdU |  | (Click-iT reaction cocktail) | ThermoFisher Scientific, C10637 | Alexa-fluor 488 |  |  |  |
| Laminin | Rabbit | 1:250 | Abcam, ab11575 | Alexa-fluor 488 donkey anti-rabbit | Jackson Immuno-Reseach, 711-545-152 |  |  |
| DAPI |  | 0.5 μg/mL | ThermoFisher Scientific, D3571 |  |  |  |  |

| **Step** | **Instructions** | **Time / Temperature** | **Materials** | **Notes** |
| --- | --- | --- | --- | --- |
| 1 | Dehydrate slides | 30 min / 60°C | Heat block |  |
| 2 | Fix | 15 min / RT | 4% PFA |  |
| 3 | Wash | 3x5 min / RT | 1X PBS |  |
| 4 | Antigen retrieval | 30 min / 90°C water bath | Citrate buffer | 900 mL ddH_2_O, 1.92 g citric acid, pH to 6.0 with NaOH, 5 mL Tween 20, top to 1000 mL |
| 5 | Antigen retrieval 2 | 30 min / RT | Citrate buffer | Take out of water bath and leave for 30 min |
| 6 | Wash | quick | 1X PBS |  |
| 7 | Permeabilize | 15 min / RT | 0.5% Triton X-100 |  |
| 8 | Wash | 3x5 min / RT | 1X PBS |  |
| 9 | Block | 1 h / RT | 0.1% Triton X-100, 5% DS, MoM block |  |
| 10 | Wash | 3x5 min / RT | 0.1% Triton X-100 |  |
| 11 | Primary antibodies | ON / 4°C | PAX7, Ki67, Laminin in block |  |
| 12 | Wash | 3x5 min / RT | 0.1% Triton X-100 |  |
| 13 | Secondary antibodies | 1 h / RT | Biotin anti-mouse 1:250, 488 anti-rat 1:2 000, 647 anti-rabbit 1:500 in 0.1% Triton X-100 | Everything conducted in the dark going forward |
| 14 | Wash | 3x5 min / RT | 0.1% Triton X-100 |  |
| 15 | Tertiary antibody | 1 h / RT | Cy3-conjugated streptavidin 1:250 in 0.1% Triton X-100 |  |
| 16 | Wash | 3x5 min / RT | 0.1% Triton X-100 |  |
| 17 | Click-iT Plus reaction cocktail | 30 min / RT | Click-iT Plus reaction cocktail | Per slide, add (in order) 440 μL 1X reaction buffer, 10 μL copper protectant, 1.2 μL Alexa Fluor picolyl azide, 45 μL ddH_2_O, 5 uL 10X reaction buffer additive |
| 18 | Wash | 3x5 min / RT | 0.1% Triton X-100 |  |
| 19 | DAPI | 5 min / RT | DAPI 1:20 000 in 1X PBS |  |
| 20 | Wash | 3x5 min / RT | 1X PBS |  |
| 21 | Dry | 15-30 min / RT |  |  |
| 22 | Mount and coverslip |  | Fluorescence mounting media and coverslip |  |

**PAX7/MYOD, EDL MYOFIBRES.**

| **Marker** | **Species** | **Dilution** | **Antibody information** | **Secondary conjugation** | **Secondary antibody information** | **Tertiary antibody** | **Tertiary antibody information** |
| --- | --- | --- | --- | --- | --- | --- | --- |
| PAX7 | Mouse | 1:100 | DSHB, PAX7-c | Biotin donkey anti-mouse | Invitrogen, 715-066-150 | Cy3-conjugated streptavidin | Jackson Immuno-Research, 016-160-084 |
| MYOD | Rabbit | 1:200 | Novus Biologicals, NBP1-54153 | Alexa-fluor 488 donkey anti-rabbit | Jackson Immuno-Research, 711-545-152 |  |  |
| DAPI |  | 0.5 μg/mL | ThermoFisher Scientific, D3571 |  |  |  |  |

| **Step** | **Instructions** | **Time / Temperature** | **Materials** | **Notes** |
| --- | --- | --- | --- | --- |
| 1 | Move fibres to 6-well plate |  | 6-well culture plate with 1X PBS | Pre-coat all wells with HS. Fibres are in suspension, so between steps, remove as much liquid as possible without removing fibres and add the next. |
| 2 | Wash | 2x5 min / 37°C | 1X PBS | Pre-warm PBS |
| 3 | Fix | 15 min / 37°C | 4% PFA |  |
| 4 | Wash | 2x5 min / RT | 1X PBS |  |
| 5 | Quench | 5 min | 100 mM glycine in 0.1% Triton X-100 |  |
| 6 | Wash | 2x5 min / RT | 0.1% Triton X-100 |  |
| 7 | Block | 3 h / RT | 5% GS, 2% BSA, 1% sodium azide in 0.2% Triton X-100 |  |
| 8 | Wash | 2x5 min / RT | 0.1% Triton X-100 |  |
| 9 | Primary antibodies | ON / 4°C | PAX7, MYOD in block | Rocking |
| 10 | Wash | 2x5 min / RT | 0.1% Triton X-100 |  |
| 11 | Biotin | 1 h / RT | Biotin anti-mouse1:250 in block | Everything conducted in the dark going forward |
| 12 | Wash | 2x5 min / RT | 0.1% Triton X-100 |  |
| 13 | Secondary/Tertiary antibodies | 1 h / RT | Cy3-conjugated streptavidin 1:250, 488 anti-rabbit 1:500 in 0.1% Triton X-100 |  |
| 14 | Wash | 2x5 min / RT | 0.1% Triton X-100 |  |
| 15 | DAPI | 2 min | DAPI 1:20 000 in 1X PBS |  |
| 16 | Wash | 3x5 min / RT | 1X PBS |  |
| 17 | Move to slides | 5-10 min / RT | Slides | Move all liquid with fibres onto slide, allow to settle, then manually aspirate liquid without aspirating fibres |
| 18 | Dry | 15-30 min / RT |  |  |
| 19 | Mount and coverslip |  | Fluorescence mounting media and coverslip |  |

**PAX7/WGA, PRIMARY SATELLITE CELLS.**

| **Marker** | **Species** | **Dilution** | **Antibody information** | **Secondary conjugation** | **Secondary antibody information** |
| --- | --- | --- | --- | --- | --- |
| PAX7 | Mouse | 1:100 | DSHB, PAX7-c | Cy3 donkey anti-mouse | Jackson Immuno-Research, 715-165-150 |
| Wheat germ agglutinin |  | 1:100 | Invitrogen, W11261 | Alexa-fluor 488 |  |
| DAPI |  | 0.5 μg/mL | ThermoFisher Scientific, D3571 |  |  |

| **Step** | **Instructions** | **Time / Temperature** | **Materials** | **Notes** |
| --- | --- | --- | --- | --- |
| 1 | Wash cells | 5 min / RT | 1X PBS |  |
| 2 | Fix | 15 min / RT | 4% PFA |  |
| 3 | Wash | 3x5 min / RT | 1X PBS |  |
| 4 | Permeabilize | 15 min / RT | 0.5% Triton X-100 |  |
| 5 | Wash | 3x5 min / RT | 1X PBS |  |
| 6 | Primary antibody | ON / 4°C | PAX7 in 0.1% Triton X-100 |  |
| 7 | Wash | 3x5 min / RT | 0.1% Triton X-100 |  |
| 8 | Secondary antibody | 1 h / RT | Cy3 anti-mouse 1:500 in 0.1% Triton X-100 | Everything conducted in the dark going forward |
| 9 | Wash | 3x5 min / RT | 0.1% Triton X-100 |  |
| 10 | DAPI + WGA | 5 min / RT | 488-conjegated WGA 1:100 in DAPI (1:20 000 in 1X PBS) |  |
| 11 | Wash | 3x5 min / RT | 1X PBS |  |
| 12 | Dry | 15-30 min / RT |  |  |
| 13 | Mount and coverslip |  | Fluorescence mounting media and coverslip |  |
