# Supplementary material for: Single‐Cell RNA‐Sequencing Reveals Cachectic Satellite Cell Population in Muscle of Male Mice With Cancer Cachexia
Source: J Cachexia Sarcopenia Muscle. 2026 Mar 27;17(2):e70260. doi: 10.1002/jcsm.70260 (PMC13140835; doi:10.1002/jcsm.70260)
Supplement: Supplementary file 22 — Table S1: Key resources. Table S2: Immunofluorescence staining antibodies. Table S3: Flow cytometry antibodies and reagents. Table S4: Magnetic beads‐conjugated antibodies for MACS. Table S5: Software and algorithms. Table S6: R packages and versions. Table S7: Complete blood cell counts. [file JCSM-17-e70260-s014.docx]

**Supplementary Table S1**: Key resources.

| **Item/material** | **Source** | **Catalogue number** | **Used for** |
| --- | --- | --- | --- |
| Basic fibroblast growth factor | ThermoFisher Scientific | 100-18B | Primary satellite cell culture |
| Bovine serum albumin | BioShop | 9048-46-8 | MACS buffer |
| Cardiotoxin | Latoxan | L8102 | Muscle injuries |
| Chamber slides (8-well) | Ibidi | 80826 | Primary satellite cell culture |
| Chicken embryo extract | Fisher Scientific | MP92850145 | Single EDL myofibre culture |
| Click-iT Plus EdU cell proliferation kit | ThermoFisher Scientific | C10637 | Histology/imaging |
| Collagenase B | Roche | 11088815001 | Enzymatic solution for muscle digestion |
| Collagenase I | MilliporeSigma | C0130 | EDL myofibre dissociation |
| Cover glass | Fisher Scientific | 12541033CA | Histology/imaging |
| DAKO pen | DAKO | S2002 | Histology/imaging |
| Dispase II | Roche | 04942078001 | Enzymatic solution for muscle digestion |
| DMEM | Wisent | 319-005-CL | Cell culture growth media |
| DNAse | Qiagen | 79256 | Enzymatic solution for muscle digestion |
| Donkey serum | Cedarlane | 007-000-121 | Histology/imaging |
| EchoMRI Body Composition Analyzer | EchoMRI | EchoMRI-700 | Body composition analysis |
| Epredia HM525 NX Cryostat | Fisher Scientific | 95-664-0EC | Muscle sectioning |
| Fetal bovine serum | Wisent | 80150 | Cell culture growth media |
| Filters (100 μm) | Corning | 352360 | Preparation of single-cell solutions |
| Filters (70 μm) | Corning | 431751 | Preparation of single-cell solutions |
| Filters (40 μm) | Corning | 431750 | Preparation of single-cell solutions |
| Filters (0.22 μm) with PES membrane | Fisher Scientific | SLGP033RS | Media and buffer filtration |
| Fluorescence mounting medium | DAKO | S3023 | Muscle, EDL, cell imaging |
| Formaldehyde | MilliporeSigma | F1635 | Histology/imaging |
| GentleMACS C tubes | Miltenyi Biotec | 130-093-237 | Muscle digestion |
| Glycine | Bio Basic | 56-40-6 | Histology/imaging |
| Goat serum | Cedarlane | 005-000-121 | Histology/imaging |
| Ham’s F-10 media | Gibco | 11550-043 | Enzymatic solution for muscle digestion |
| Hepatocyte growth factor | ThermoFisher Scientific | 100-39 | Primary satellite cell culture |
| Horse serum | MilliporeSigma | H1138 | Primary satellite cell differentiation media |
| Isopentane | Fisher Scientific | AC126470025 | Histology/imaging |
| LD columns | Miltenyi Biotec | 130-042-901 | MACS |
| Lewis Lung Carcinoma cells | ATCC | CRL-1642 | Tumour inoculations |
| LS columns | Miltenyi Biotec | 130-042-101 | MACS |
| MACS stand | Miltenyi Biotech | 130-042-303 | MACS |
| Matrigel | VWR | CACB354234 | Primary satellite cell culture |
| Methanol | ThermoFisher Scientific | 031721.K7 | Cell staining |
| Microvette tubes | Sarstedt | 16.444.100 | Blood collection |
| O.C.T. | Fisher Scientific | 4585 | Histology/imaging |
| OctoMACS dissociator | Miltenyi Biotec | 130-096-427 | Muscle digestion |
| Penicillin/streptomycin | Wisent | 450-201-EL | Cell culture growth media |
| QuadroMACS separator | Miltenyi Biotec | 130-090-976 | MACS |
| Red Blood Cell Lysis Buffer | MilliporeSigma | R7757 | Preparation of single-cell solutions |
| Senescence β-Galactosidase staining kit | Cell Signaling Technology | 9860S | Histology/imaging |
| Superfrost Plus Microscope slides | Fisher Scientific | 1255015 | Histology/imaging |
| Triton-X 100 | BioShop | TRX777 | Histology/imaging |
| Tumour dissociation kit | Miltenyi Biotec | 130-096-730 | Tumour digestion |
| Tween 20 | Fisher Scientific | 9005-64-5 | Histology/imaging |

**Supplementary Table S2**: Immunofluorescence staining antibodies.

| **Marker** | **Type** | **Dilution** | **Source** | **Catalogue #** | **Used for** |
| --- | --- | --- | --- | --- | --- |
| DAPI |  | 0.5 μg/mL | Thermo-Fisher Scientific | D3571 | Nuclear staining |
| Ki67 | Primary | 1:200 | Invitrogen | 14-5698-82 | Muscle sections |
| Laminin | Primary | 1:250 | Abcam | Ab11575 | Muscle sections |
| MF20 | Primary | 1:50 | DSHB | MF20-s | Satellite cells *ex vivo* differentiation |
| MYOD | Primary | 1:100 | Novus Biologicals | NBP1-54153 | EDL myofibres |
| PAX7 | Primary | 1:100 | DSHB | PAX7-c | Satellite cells in muscle sections, *ex vivo*, EDL myofibres |
| Wheat germ agglutinin (488 conjugated) | Primary + Secondary | 1:100 | Invitrogen | W11261 | Muscle sections, satellite cell *ex vivo* |
| Biotin donkey anti-mouse | Secondary | 1:250 | Jackson Immuno-Research | 715-066-150 | PAX7 staining in muscle sections |
| Cy3 donkey anti-mouse | Secondary | 1:500 | Jackson Immuno-Research | 715-165-150 | PAX7, MF20 |
| 488 donkey anti-rat | Secondary | 1:2 000 | Invitrogen | A21208 | Ki67 |
| 488 donkey anti-rabbit | Secondary | 1:500 | Jackson Immuno-Research | 711-545-152 | Laminin, MYOD |
| 647 goat anti-rabbit | Secondary | 1:500 | Jackson Immuno-Research | 111-605-003 | Laminin |
| Cy3-conjugated streptavidin | Tertiary | 1:250 | Jackson Immuno-Research | 016-160-084 | Biotin-bound PAX7 |

**Supplementary Table S3**: Flow cytometry antibodies and reagents.

| **Marker/ application** | **Fluorophore** | **Dilution** | **Source** | **Catalogue #** | | **Used for** |
| --- | --- | --- | --- | --- | --- | --- |
| Compensation beads | n/a | 1 μL antibody/ drop | ThermoFisher Scientific | 01-2222-41 | Compensation | |
| True-stain monocyte block | n/a | 1:200 | BioLegend | 426102 | Flow cytometry, immune populations | |
| Fc-receptor block | n/a | 1:100 | Miltenyi Biotec | 130-092-575 | Flow cytometry, immune populations | |
| Calcein AM | n/a | 1:1000 | ThermoFisher Scientific | C3100-MP | Live/Dead FACS scRNAseq | |
| SYTOX^TM^ AADvanced^TM^ Dead stain kit | 7AAD | 1:1000 | ThermoFisher Scientific (Invitrogen) | S10349 | Live/Dead FACS scRNAseq; Flow cytometry muscle populations | |
| CD31 | BV421 | 1:200 | BioLegend | 102423 | Flow cytometry muscle populations | |
| CD45 | BV785 | 1:200 | BioLegend | 103149 | Flow cytometry muscle populations | |
| ITGA7 | APC | 1:100 | R&D Systems | FAB3518A | Flow cytometry muscle populations | |
| PDGFRα | BV605 | 1:100 | BioLegend | 135916 | Flow cytometry muscle populations | |
| LIVE/DEAD^TM^ Blue | n/a | 1:1000 | ThermoFisher Scientific (Invitrogen) | L23105 | Flow cytometry, immune populations | |
| CD45 | FITC | 1:200 | BioLegend | 103107 | Flow cytometry, immune populations | |
| CD11b | BV605 | 1:200 | BioLegend | 101237 | Flow cytometry, immune populations | |
| CD11c | BV480 | 1:200 | BD BioSciences | 565627 | Flow cytometry, immune populations | |
| Ly6C | APC-Cy7 | 1:200 | BD BioSciences | 560596 | Flow cytometry, immune populations | |
| Ly6G | BV421 | 1:200 | BioLegend | 127627 | Flow cytometry, immune populations | |
| F4/80 | APC | 1:200 | BioLegend | 123115 | Flow cytometry, immune populations | |
| CD206 | PE | 1:200 | BioLegend | 141705 | Flow cytometry, immune populations | |
| CD3e | Per-CP | 1:100 | BD BioSciences | 553067 | Flow cytometry, immune populations | |
| CD19 | AF700 | 1:200 | BD BioSciences | 560515 | Flow cytometry, immune populations | |
| NK1.1 | BV786 | 1:200 | BD BioSciences | 563333 | Flow cytometry, immune populations | |

NOTES – True-Stain monocyte and Fc-receptor blocks were incubated for 10 minutes prior to cell staining, then proceeded directly to cell staining. When staining for the muscle populations, PDGFRα was stained first, washed, then proceeded to staining the other markers. Antibodies were stained for 30 minutes on ice and in the dark, washed, then stained for respective live/dead stains. Samples for FACS and muscle preparations were stained in FACS buffer, where immune cell panels in the muscle, spleen, and tumour were stained in PBS due to LIVE/DEAD Blue binding to free amines in solution.

**Supplementary Table S4**: Magnetic beads-conjugated antibodies for MACS.

| **Marker** | **Dilution** | **Catalogue #** |
| --- | --- | --- |
| CD31 | 1:10 | 130-097-418 |
| CD45 | 1:10 | 130-052-301 |
| PDGFRα | 1:10 | 130-101-502 |
| ITGA7 | 1:2.5 | 130-104-261 |

All antibodies are from Miltenyi Biotec.

**Supplementary Table S5**: Software and algorithms.

| Software/algorithm | Version | Company |
| --- | --- | --- |
| BioRender | n/a | biorender.com |
| CellRanger | 5.0.0 | 10X Genomics |
| FIJI | 2.16.0/1.54p | Open source |
| FlowJo | 10.10.0 | BD Biosciences |
| GitHub | n/a | Github.com |
| Illustrator | 29.7.1 | Adobe |
| MIRA Vision | 0.0.1 | mira-vision.com |
| Prism | 10.5.0 | GraphPad |
| R | 4.4.1 | Open source |
| R Studio | 2024.09.0+375 | Open source |
| SpectroFlo | 3.0.1 | Cytek Biosciences |
| ZEN Pro Imaging | 3.1 | Zeiss |

**Supplementary Table S6**: R packages and versions.

| **Package** | **Version** | **Repository** | **Reference** |
| --- | --- | --- | --- |
| AnnotationDbi | 1.66.0 | BioConductor 3.19 | Pagès H, Carlson M, Falcon S, Li N. AnnotationDbi: Manipulation of SQLite‑based annotations in Bioconductor. R package, 2024. doi:10.18129/B9.bioc.AnnotationDbi. |
| base | 4.4.1 | R | R Core Team. R: A language and environment for statistical computing. R Foundation for Statistical Computing, Vienna, Austria, 2024. [https://www.R-project.org/](https://www.r-project.org/). |
| Biobase | 2.64.0 | BioConductor 3.19 | Huber W, Carey VJ, Gentleman R, Anders S, Carlson M, Carvalho BS, et al. Orchestrating high-throughput genomic analysis with Bioconductor. Nat Methods 2015;12:115–121. doi:10.1038/nmeth.3252. |
| BiocGenerics | 0.50.0 | BioConductor 3.19 | Huber W, Carey VJ, Gentleman R, Anders S, Carlson M, Carvalho BS, et al. Orchestrating high-throughput genomic analysis with Bioconductor. Nat Methods 2015;12:115–121. doi:10.1038/nmeth.3252. |
| CellChat | 2.1.2 | GitHub (CellChat) | Jin S. CellChat: Inference and analysis of cell‑cell communication from single‑cell and spatially resolved transcriptomics data. R package version 2.1.2, 2024. |
| circlize | 0.4.16 | CRAN | Gu Z. circlize implements and enhances circular visualization in R. Bioinformatics 2014;30:2811–2812. doi:10.1093/bioinformatics/btu393. |
| cluster | 2.1.6 | CRAN | Maechler M, Rousseeuw P, Struyf A, Hubert M, Hornik K. cluster: Cluster analysis basics and extensions. R package version 2.1.6, 2023. |
| ClusterProfiler | 4.12.6 | BioConductor 3.19 | Gu Z. Complex heatmaps reveal patterns and correlations in multidimensional genomic data. Bioinformatics 2016;32:2847–2849. doi:10.1093/bioinformatics/btw313.  Gu Z. Complex Heatmap Visualization. iMeta 2022. |
| ComplexHeatmap | 2.20.0 | BioConductor 3.19 | Gu Z, Eils R, Schlesner M. Complex heatmaps reveal patterns and correlations in multidimensional genomic data. Bioinformatics 2016;32:2847–2849. doi:10.1093/bioinformatics/btw313. |
| cowplot | 1.1.3 | CRAN | Wilke C. cowplot: Streamlined plot theme and plot annotations for 'ggplot2'. R package version 1.1.3, 2024. [https://CRAN.R-project.org/package=cowplot](https://cran.r-project.org/package=cowplot). |
| datasets | 4.4.1 | R | R Core Team. R: A language and environment for statistical computing. R Foundation for Statistical Computing, Vienna, Austria, 2024. [https://www.R-project.org/](https://www.r-project.org/). |
| data.table | 1.16.2 | CRAN | Barrett T, Dowle M, Srinivasan A, Gorecki J, Chirico M, Hocking T, Schwendinger B. data.table: Extension of data.frame. R package version 1.16.2, 2024. [https://CRAN.R-project.org/package=data.table](https://cran.r-project.org/package=data.table). |
| DDRTree | 0.1.5 | CRAN | Qiu X, Trapnell C, Mao Q, Wang L. DDRTree: Learning Principal Graphs with DDRTree. R package version 0.1.5, 2017. |
| dplyr | 1.1.4 | CRAN | Wickham H, François R, Henry L, Müller K, Vaughan D. dplyr: A grammar of data manipulation. R package version 1.1.4, 2023. [https://CRAN.R-project.org/package=dplyr](https://cran.r-project.org/package=dplyr). |
| EnhancedVolcano | 1.22.0 | BioConductor 3.19 | Blighe K, Rana S, Lewis M. EnhancedVolcano: Publication‑ready volcano plots with enhanced colouring and labeling. R package version 1.22.0, 2024. doi:10.18129/B9.bioc.EnhancedVolcano. |
| forcats | 1.0.0 | CRAN | Wickham H. forcats: Tools for working with categorical variables (factors). R package version 1.0.0, 2023. [https://CRAN.R-project.org/package=forcats](https://cran.r-project.org/package=forcats). |
| GenomeInfoDb | 1.40.1 | BioConductor 3.19 | Arora S, Morgan M, Carlson M, Pagès H. GenomeInfoDb: Utilities for manipulating chromosome names, including modifying them to follow a particular naming style. R package version 1.40.1, 2024. doi:10.18129/B9.bioc.GenomeInfoDb. |
| GenomicRanges | 1.56.2 | BioConductor 3.19 | Lawrence M, Huber W, Pagès H, Aboyoun P, Carlson M, et al. Software for computing and annotating genomic ranges. PLoS Comput Biol 2013;9:e1003118. doi:10.1371/journal.pcbi.1003118. |
| ggbeeswarm | 0.7.2 | CRAN | Clarke E, Sherrill-Mix S, Dawson C. ggbeeswarm: Categorical scatter (violin point) plots. R package version 0.7.2, 2023. [https://CRAN.R-project.org/package=ggbeeswarm](https://cran.r-project.org/package=ggbeeswarm). |
| ggplot2 | 3.5.1 | CRAN | Wickham H. ggplot2: Elegant graphics for data analysis. New York: Springer-Verlag; 2016. |
| ggrepel | 0.9.6 | CRAN | Slowikowski K (2024). ggrepel: Automatically Position Non Overlapping Text Labels with 'ggplot2'. R package version  0.9.6,  <https://CRAN.R-project.org/package=ggrepel>. |
| ggvenn | 0.1.10 | CRAN | Yan L (2023). Ggvenn: Draw Venn Diagram by ‘ggplot2’. R package version 0.1.10, <https://CRAN.R-project.org/package=ggvenn>. |
| graphics | 4.4.1 | R | R Core Team. R: A language and environment for statistical computing. R Foundation for Statistical Computing, Vienna, Austria, 2024. [https://www.R-project.org/](https://www.r-project.org/). |
| grDevices | 4.4.1 | R | R Core Team. R: A language and environment for statistical computing. R Foundation for Statistical Computing, Vienna, Austria, 2024. [https://www.R-project.org/](https://www.r-project.org/). |
| grid | 4.4.1 | R | R Core Team. R: A language and environment for statistical computing. R Foundation for Statistical Computing, Vienna, Austria, 2024. [https://www.R-project.org/](https://www.r-project.org/). |
| gridExtra | 2.3 | CRAN | Auguie B. gridExtra: Miscellaneous functions for "grid" graphics. R package version 2.3, 2017. [https://CRAN.R-project.org/package=gridExtra](https://cran.r-project.org/package=gridExtra). |
| harmony | 1.2.3 | CRAN | Korsunsky I, Hemberg M, Patikas N, Yao H, Millard N, Fan J, Slowikowski K, Raychaudhuri S. harmony: Fast, sensitive, and accurate integration of single cell data. R package version 1.2.3, 2024. [https://CRAN.R-project.org/package=harmony](https://cran.r-project.org/package=harmony). |
| hdf5r | 1.3.12 | CRAN | Hoefling H, Annau M. hdf5r: Interface to the 'HDF5' binary data format. R package version 1.3.12, 2025. [https://CRAN.R-project.org/package=hdf5r](https://cran.r-project.org/package=hdf5r). |
| igraph | 2.0.3 | CRAN | Csardi G, Nepusz T. The igraph software package for complex network research. InterJournal Complex Systems 2006;1695. [https://igraph.org](https://igraph.org/). |
| IRanges | 2.38.1 | BioConductor 3.19 | Lawrence M, Huber W, Pagès H, Aboyoun P, Carlson M, et al. Software for computing and annotating genomic ranges. PLoS Comput Biol 2013;9:e1003118. doi:10.1371/journal.pcbi.1003118. |
| irlba | 2.3.5.1 | CRAN | Baglama J, Reichel L, Lewis B. irlba: Fast truncated singular value decomposition and principal components analysis for large dense and sparse matrices. R package version 2.3.5.1, 2022. [https://CRAN.R-project.org/package=irlba](https://cran.r-project.org/package=irlba). |
| lubridate | 1.9.3 | CRAN | Grolemund G, Wickham H. Dates and times made easy with lubridate. J Stat Softw 2011;40:1–25. <https://www.jstatsoft.org/v40/i03/>. |
| Matrix | 1.7.0 | CRAN | Bates D, Maechler M, Jagan M. Matrix: Sparse and dense matrix classes and methods. R package version 1.7-0, 2024. [https://CRAN.R-project.org/package=Matrix](https://cran.r-project.org/package=Matrix). |
| MatrixGenerics | 1.16.0 | BioConductor 3.19 | Ahlmann-Eltze C, Hickey P, Pagès H. MatrixGenerics: S4 generic summary statistic functions that operate on matrix-like objects. R package version 1.16.0, 2024. doi:10.18129/B9.bioc.MatrixGenerics. |
| matrixStats | 1.4.1 | CRAN | Bengtsson H. matrixStats: Functions that apply to rows and columns of matrices (and to vectors). R package version 1.4.1, 2024. [https://CRAN.R-project.org/package=matrixStats](https://cran.r-project.org/package=matrixStats). |
| methods | 4.4.1 | R | R Core Team. R: A language and environment for statistical computing. R Foundation for Statistical Computing, Vienna, Austria, 2024. [https://www.R-project.org/](https://www.r-project.org/). |
| monocle3 | 1.3.5 | GitHub (monocle3) | Trapnell C, Cacchiarelli D, Grimsby J, Pokharel P, Li S, Morse M, et al. The dynamics and regulators of cell fate decisions are revealed by pseudo-temporal ordering of single cells. Nat Biotechnol 2014;32:381–386. doi:10.1038/nbt.2859.  Qiu X, Hill A, Packer J, Lin D, Ma YA, Trapnell C. Single-cell mRNA quantification and differential analysis with Census. Nat Methods 2017;14:309–315. doi:10.1038/nmeth.4150.  Qiu X, Mao Q, Tang Y, Wang L, Chawla R, Pliner HA, et al. Reverse graph embedding resolves complex single-cell developmental trajectories. Nat Methods 2017;14:979–982. doi:10.1038/nmeth.4402.  Cao J, Spielmann M, Qiu X, Huang X, Ibrahim DM, Hill AJ, et al. The single-cell transcriptional landscape of mammalian organogenesis. Nature 2019;566:496–502. doi:10.1038/s41586-019-0969-x. |
| msigdbr | 10.0.1 | CRAN | Dolgalev I. msigdbr: MSigDB gene sets for multiple organisms in a tidy data format. R package version 10.0.1, 2025. [https://CRAN.R-project.org/package=msigdbr](https://cran.r-project.org/package=msigdbr). |
| NMF | 0.28 | CRAN | Gaujoux R, Seoighe C. A flexible R package for nonnegative matrix factorization. BMC Bioinformatics 2010;11:367. doi:10.1186/1471-2105-11-367. |
| org.Mm.eg.db | 3.19.1 | BioConductor 3.19 | Carlson M. org.Mm.eg.db: Genome wide annotation for Mouse. R package version 3.19.1, 2024. |
| patchwork | 1.3.0 | CRAN | Pedersen, TL. patchwork: The Composer of Plots. R package version 1.3.0. Vienna, Austria: R Foundation for Statistical Computing; 2024. <http://CRAN.R-project.org/package=patchwork>. |
| pheatmap | 1.0.12 | CRAN | Kolde R. pheatmap: Pretty heatmaps. R package version 1.0.12, 2019. [https://CRAN.R-project.org/package=pheatmap](https://cran.r-project.org/package=pheatmap). |
| presto | 1.0.0 | GitHub (presto) | Korsunsky I, Nathan A, Millard N, Raychaudhuri S. presto: Fast functions for differential expression using Wilcox and AUC. R package version 1.0.0, 2024. <https://github.com/immunogenomics/presto>. |
| purrr | 1.0.2 | CRAN | Wickham H, Henry L. purrr: Functional programming tools. R package version 1.0.2, 2023. [https://CRAN.R-project.org/package=purrr](https://cran.r-project.org/package=purrr). |
| RColorBrewer | 1.1.3 | CRAN | Neuwirth E. RColorBrewer: ColorBrewer palettes. R package version 1.1-3, 2022. [https://CRAN.R-project.org/package=RColorBrewer](https://cran.r-project.org/package=RColorBrewer). |
| Rcpp | 1.0.13 | CRAN | Eddelbuettel D, Francois R, Allaire J, Ushey K, Kou Q, Russell N, Ucar I, Bates D, Chambers J. Rcpp: Seamless R and C++ integration. R package version 1.0.13, 2024. [https://CRAN.R-project.org/package=Rcpp](https://cran.r-project.org/package=Rcpp). |
| readr | 2.1.5 | CRAN | Wickham H, Hester J, Bryan J. readr: Read rectangular text data. R package version 2.1.5, 2024. [https://CRAN.R-project.org/package=readr](https://cran.r-project.org/package=readr). |
| registry | 0.5.1 | CRAN | Eyer D. registry: Infrastructure for R package registries. R package version 0.5-1, 2019. [https://CRAN.R-project.org/package=registry](https://cran.r-project.org/package=registry). |
| rngtools | 1.5.2 | CRAN | Gaujoux R. rngtools: Utility functions for working with random number generators. R package version 1.5.2, 2021. [https://CRAN.R-project.org/package=rngtools](https://cran.r-project.org/package=rngtools). |
| scDblFinder | 1.18.0 | BioConductor 3.19 | Germain P, Lun A, Garcia Meixide C, Macnair W, Robinson M. Doublet identification in single-cell sequencing data using scDblFinder. F1000Res 2022. doi:10.12688/f1000research.73600.2. |
| Seurat | 5.0.3 | CRAN | Hao Y, Hao S, Andersen-Nissen E, Mauck WM 3rd, Zheng S, Butler A, Lee MJ, Wilk AJ, Darby C, Zager M, et al. Dictionary learning for integrative, multimodal and scalable single-cell analysis. Nat Biotechnol 2023. doi:10.1038/s41587-022-01585-1. |
| SeuratObject | 5.0.2 | CRAN | Hoffman P, Satija R, Collins D, Hao Y, Hartman A, Molla G, Butler A, Stuart T. SeuratObject: Data structures for single cell data. R package version 5.0.2, 2024. [https://CRAN.R-project.org/package=SeuratObject](https://cran.r-project.org/package=SeuratObject). |
| SeuratWrappers | 0.3.1 | GitHub (SeuratWrappers) | Satija R, Butler A, Hoffman P, Stuart T. SeuratWrappers: Community-provided methods and extensions for the Seurat object. R package version 0.3.1, 2022. <https://github.com/satijalab/seurat-wrappers>. |
| SingleCellExperiment | 1.26.0 | BioConductor 3.19 | Amezquita RA, Lun AT, Becht E, Carey VJ, Carpp LN, Geistlinger L, Marini F, Rue-Albrecht K, Risso D, Soneson C, Waldron L, Pagès H, Smith M, Huber W, Morgan M, Gottardo R, Hicks SC. Orchestrating single-cell analysis with Bioconductor. Nat Methods 2020;17:137–145. doi:10.1038/s41592-019-0654-x. |
| sp | 2.2.0 | CRAN | Pebesma E, Bivand R. Classes and methods for spatial data in R. R News 2005;5(2):9–13. [https://CRAN.R-project.org/doc/Rnews/](https://cran.r-project.org/doc/Rnews/).  Bivand R, Pebesma E, Gomez-Rubio V. Applied spatial data analysis with R, second edition. New York: Springer; 2013. <https://asdar-book.org/>. |
| stats | 4.4.1 | R | R Core Team. R: A language and environment for statistical computing. R Foundation for Statistical Computing, Vienna, Austria, 2024. [https://www.R-project.org/](https://www.r-project.org/). |
| stats4 | 4.4.1 | R | R Core Team. R: A language and environment for statistical computing. R Foundation for Statistical Computing, Vienna, Austria, 2024. [https://www.R-project.org/](https://www.r-project.org/). |
| stringr | 1.5.1 | CRAN | Wickham H. stringr: Simple, consistent wrappers for common string operations. R package version 1.5.1, 2023. [https://CRAN.R-project.org/package=stringr](https://cran.r-project.org/package=stringr). |
| SummarizedExperiment | 1.34.0 | BioConductor 3.19 | Morgan M, Obenchain V, Hester J, Pagès H. SummarizedExperiment: SummarizedExperiment container. R package version 1.34.0, 2024. doi:10.18129/B9.bioc.SummarizedExperiment  . |
| S4Vectors | 0.42.1 | BioConductor 3.19 | Pagès H, Lawrence M, Aboyoun P. S4Vectors: Foundation of vector-like and list-like containers in Bioconductor. R package version 0.42.1, 2024. doi:10.18129/B9.bioc.S4Vectors. |
| tibble | 3.2.1 | CRAN | Müller K, Wickham H. tibble: Simple data frames. R package version 3.2.1, 2023. [https://CRAN.R-project.org/package=tibble](https://cran.r-project.org/package=tibble). |
| tidyr | 1.3.1 | CRAN | Wickham H, Vaughan D, Girlich M. tidyr: Tidy messy data. R package version 1.3.1, 2024. [https://CRAN.R-project.org/package=tidyr](https://cran.r-project.org/package=tidyr). |
| tidyverse | 2.0.0 | CRAN | Wickham H, Averick M, Bryan J, Chang W, McGowan LD, François R, Grolemund G, Hayes A, Henry L, Hester J, Kuhn M, Pedersen TL, Miller E, Bache SM, Müller K, Ooms J, Robinson D, Seidel DP, Spinu V, Takahashi K, Vaughan D, Wilke C, Woo K, Yutani H. Welcome to the tidyverse. J Open Source Softw 2019;4:1686. doi:10.21105/joss.01686. |
| UCell | 2.11.1 | GitHub (UCell) | Andreatta M, Carmona SJ. UCell: Robust and scalable single-cell gene signature scoring. Comput Struct Biotechnol J 2021;19:3796–3798. doi:10.1016/j.csbj.2021.06.043. |
| utils | 4.4.1 | R | R Core Team. R: A language and environment for statistical computing. R Foundation for Statistical Computing, Vienna, Austria, 2024. [https://www.R-project.org/](https://www.r-project.org/). |
| viridis | 0.6.5 | CRAN | Garnier S, Ross N, Rudis R, Camargo AP, Sciaini M, Scherer C. viridis(Lite): Colorblind-friendly color maps for R. R package version 0.6.5, 2024. |
| viridisLite | 0.4.2 | CRAN | Garnier S, Ross N, Rudis R, Camargo AP, Sciaini M, Scherer C. viridisLite: Colorblind-friendly color maps for R. R package version 0.4.2, 2023. |

**Supplementary Table S7**: Complete blood cell counts.

| **Measurement** | **Sham** | **2-weeks** | **2.5-weeks** | **3.5-weeks** |
| --- | --- | --- | --- | --- |
| Neutrophil (%) | 9.5 ± 8.4 | 9.4 ± 7.7 | 18.9 ± 6.0 | **44.7 ± 9.7**** #### ††††** |
| Neutrophil (/μL) | 559.4 ± 856.5 | 406.3 ± 183.1 | 1450.2 ± 835.5 | **5966.7 ± 5520.9* # †** |
| Reticulocyte (%) | 3.0 ± 0.39 | 3.2 ± 0.57 | 3.6 ± 1.38 | **6.8 ± 3.0* # †** |
| WBC (K/μL) | 4.2 ± 2.62 | 5.3 ± 1.77 | 7.3 ± 2.59 | 13.1 ± 10.3 |
| Absolute Reticulocyte (K/μL) | 272.4 ± 28.0 | 237.3 ± 48.2 | 311.7 ± 155.8 | 506.3 ± 284.7 |
| Band (%) | 0 | 0 | 0 | 0 |
| Band (/μL) | 0 | 0 | 0 | 0 |
| RBC (M/μL) | 9.2 ± 0.9 | 7.0 ± 1.7 | 8.2 ± 2.4 | 7.4 ± 1.7 |
| Reticulocyte Hemoglobin Content (pg) | 17.4 ± 0.3 | 17.1 ± 0.3 | 17.0 ± 0.4 | 16.8 ± 0.4 |
| HGB (g/dL) | 13.4 ± 1.4 | 10.9 ± 3.8 | 13.9 ± 3.3 | 10.8 ± 2.0 |
| Lymphocytes (%) | 78.3 ± 6.4 | 74.5 ± 1.6 | 74.6 ± 6.9 | **52.0 ± 7.8**** ### ††††** |
| Lymphocyte (/μL) | 3214.6 ± 1890.9 | 3981.0 ± 1351.1 | 5386.3 ± 1931.6 | 6581.0 ± 4758.7 |
| Nucleated RBC (/100 WBC) | 0 | 0 | 0 | 0 |
| Monocytes (%) | 7.6 ± 7.1 | 11.5 ± 7.2 | 4.9 ± 4.4 | 3.1 ± 4.1 |
| Monocyte (/μL) | 278.2 ± 270.1 | 689.3 ± 458.1 | 322.9 ± 216.5 | 468.5 ± 789.0 |
| Polychromasia | Slight | Slight-Moderate | Slight | Slight |
| HCT (%) | 45.8 ± 4.5 | 36.8 ± 8.7 | 43.3 ± 12.6 | 39.4 ± 8.3 |
| Anisocytosis | Slight | Slight-Moderate | Slight | Slight |
| Eosinophils (%) | 3.9 ± 3.2 | 3.9 ± 2.2 | 1.5 ± 2.2 | **0.2 ± 0.5*** |
| Eosinophil (/μL) | 129.8 ± 136.2 | 205.0 ± 152.2 | 83.5 ± 106.1 | 33.5 ± 82.1 |
| MCV (fL) | 50.0 ± 0.7 | 52.8 ± 2.1 | **52.9 ± 1.7*** | **53.7 ± 2.4*** |
| Basophils (%) | 0.6 ± 0.5 | 0.7 ± 0.8 | - 1. ± 0.1 | - 1. ± 0.1 |
| Basophils (/μL) | 18.2 ± 13.1 | 44.0 ± 47.8 | 7.2 ± 8.4 | 9.2 ± 22.5 |
| MCH (pg) | 14.5 ± 0.3 | 15.4 ± 3.2 | 17.9 ± 4.9 | 10.3 ± 8.0 |
| Poikilocytosis | Absent | Absent | Absent | Absent |
| Heinz bodies | Absent | Absent | Absent | Absent |
| MCHC (g/dL) | 29.1 ± 0.4 | 29.3 ± 6.9 | 33.9 ± 8.9 | 27.6 ± 1.8 |
| Myelocyte (%) | 0 | 0 | 0 | 0 |
| Myelocyte (/μL) | 0 | 0 | 0 | 0 |
| Platelet Count (K/uL) | 220.4 ± 306.9 | 103.3 ± 47.4 | 436.9 ± 251.8 | 361.0 ± 337.9 |
| Promyelocyte (%) | 0 | 0 | 0 | 0 |
| Promyelocyte (/μL) | 0 | 0 | 0 | 0 |

Data are represented by means ± standard deviation or descriptions where applicable. Means were compared using a 1-way ANOVA with Tukey’s post-hoc testing. * Different compared to Sham, # different compared to 2-weeks, † different compared to 2.5-weeks where p<0.05. **/##/†† p<0.01. ***/###/††† p<0.001. ****/####/†††† p<0.0001.
